# Exploring the pharmacokinetics and tolerability of cyclooxygenase inhibitor ampiroxicam: a phase I study on single and multiple oral doses

**DOI:** 10.3389/fphar.2024.1429971

**Published:** 2024-06-21

**Authors:** Pengfei Zhao, Ying Qi

**Affiliations:** ^1^ Department of Pharmacology, School of Pharmacy, China Medical University, Shenyang, China; ^2^ Department of Radiology, Shengjing Hospital of China Medical University, Shenyang, China

**Keywords:** ampiroxicam, piroxicam, UPLC, pharmacokinetics, tolerability

## Abstract

**Introduction:** Ampiroxicam is a long-acting, non-steroidal anti-inflammatory drug that selectively inhibits human cyclooxygenase, effectively mitigating fever, pain, and inflammation. This study evaluated the drug's tolerability and pharmacokinetics to support personalized dosing strategies.

**Methods:** The study involved healthy participants and focused on the pharmacokinetics of ampiroxicam. Plasma levels of piroxicam, a key metabolite of ampiroxicam, were measured using ultra-performance liquid chromatography. Piroxicam was chosen due to its integral role in ampiroxicam's metabolic pathway. The analytical method underwent rigorous validation to ensure precision and accuracy, addressing potential interference from endogenous plasma substances.

**Results:** Participants received ampiroxicam in single doses (low, medium, and high) and multiple doses. Pharmacokinetic parameters, including AUC_0–216_, AUC_0–∞_, and C_max_, exhibited a dose-dependent increase. No significant differences were noted across the dosage groups, and sex-specific differences were minimal, with the exception of mean residence time (MRT) in the multiple-dose group, which appeared influenced by body weight variations.

**Discussion:** The findings affirm the safety and efficacy of ampiroxicam across different dosing regimens, validating its clinical utility and potential for personalized medicine in the treatment of pain and inflammation.

## Introduction

Ampiroxicam, depicted in Supplementary Figure S1A, stands as a long-acting non-steroidal anti-inflammatory drug (NSAID). It is prescribed to alleviate fever, pain, and inflammation linked to various conditions, including trauma, surgery, dental discomfort, and chronic diseases such as rheumatoid arthritis, osteoarthritis, lower back pain, shoulder periarthritis, and cervical-shoulder-wrist syndrome (Carty et al., 1993; Sai et al., 2001; Kelley et al., 2015; Kurita et al., 1991). The mechanisms of these pharmacological actions are primarily through the inhibition of cyclooxygenase, thereby reducing the synthesis of prostaglandins (PG) in the body. NSAIDs can also exert a potent and irreversible inhibitory effect on platelet aggregation by inhibiting cyclooxygenase, leading to an increased risk of bleeding in patients after medication (Gurpinar et al., 2014; Schjerning Olsen et al., 2015). Additionally, several studies have demonstrated the antitumor effects of some NSAIDs (Davidson et al., 2014; Shiff and Rigas, 1999; Husain et al., 2002). Furthermore, studies have shown that ampiroxicam exhibits antitumor properties (Gordon et al., 2015; Goto et al., 2011). The antitumor action, besides being related to the inhibition of PG production, is also associated with the activation of caspase-3 and caspase-9, induction of tumor cell apoptosis, inhibition of tumor cell proliferation, and anti-angiogenesis. Moreover, NSAIDs have been shown to prevent and delay the onset of Alzheimer’s disease (Vlad et al., 2008), treat ophthalmic diseases (Kim et al., 2010), and prevent preterm labor (Loudon et al., 2003). These findings increase the possibility of ampiroxicam as an emerging novel drug in clinical treatment in the future. Following oral administration, ampiroxicam is absorbed by the gastrointestinal tract and converted to piroxicam. Piroxicam is the primary active component of ampiroxicam and exerts anti-inflammatory effects by inhibiting cyclooxygenase, reducing prostaglandin biosynthesis, and suppressing leukocyte chemotaxis. Moreover, ampiroxicam effectively alleviates inflammatory pain responses without evidence of drug resistance while mitigating gastrointestinal irritation associated with piroxicam (Falkner et al., 1990; Rabasseda and Hopkins, 1994). Compared to other NSAIDs, ampiroxicam demonstrates superior anti-inflammatory and analgesic effects while exhibiting a lower propensity for ulcer-related complications and fewer adverse reactions during use. This highlights ampiroxicam as a potent medication with a good safety profile and minimal side effects (Munehasu et al., 1992).

**FIGURE 1:**
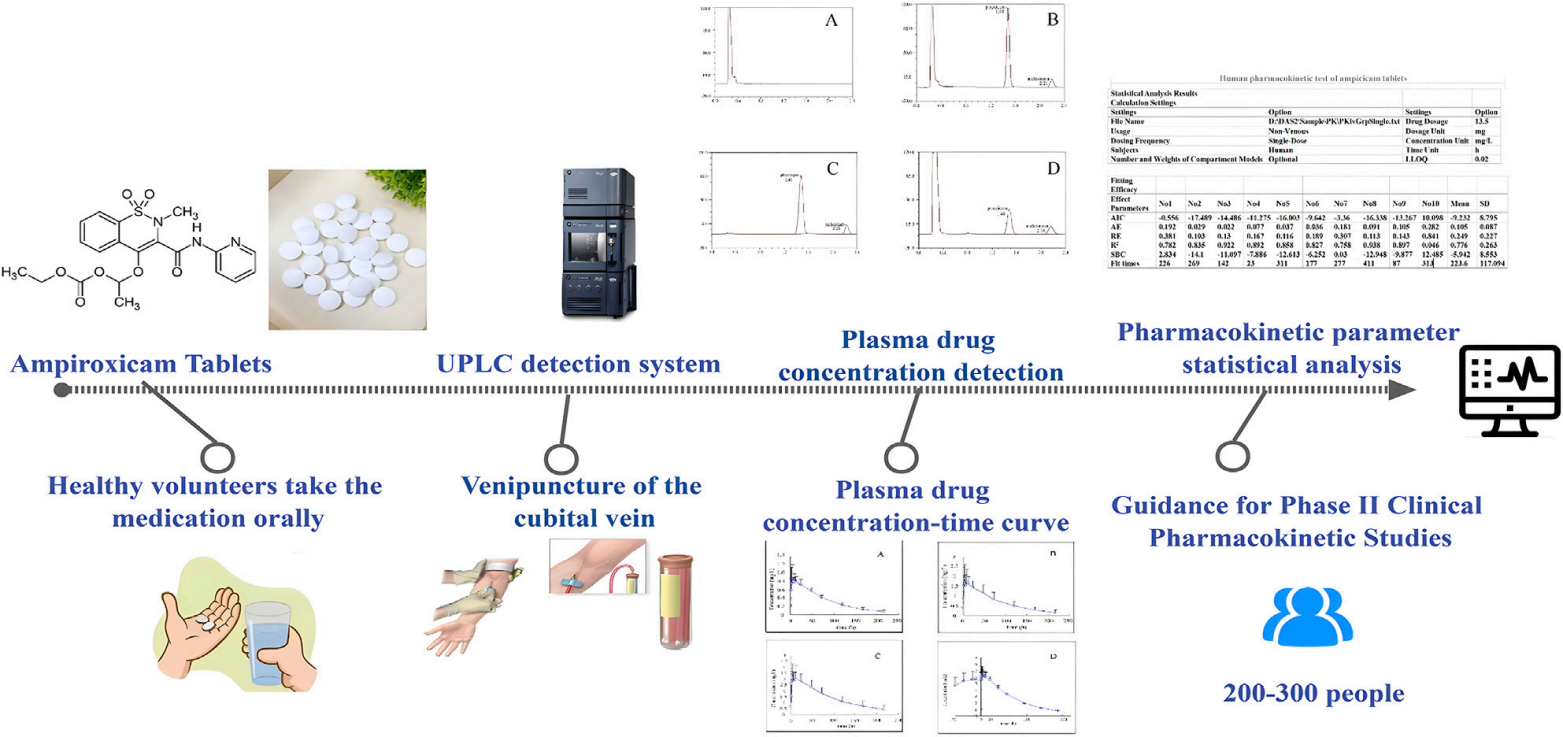
Illustration of the entire research process.

NSAIDs are currently the most widely prescribed medications in clinical settings to manage fever, alleviate pain, and reduce inflammation. Although NSAIDs offer relief to millions of patients, they also entail avoidable hardships and impose a substantial economic burden. In the United States, severe gastrointestinal side effects associated with traditional NSAIDs lead to tens of thousands of hospitalizations and fatalities annually (Cryer, 2005). In the United Kingdom, researchers have estimated that common NSAIDs prescriptions lead to approximately 12,000 hospital admissions each year, resulting in 2,230 fatalities [Blower et al., 1997]. The use of NSAIDs is substantially constrained in clinical practice because of their prominent adverse effects, mainly related to gastrointestinal issues such as ulcers, bleeding, and gastric perforations, which constitute one-third of all adverse drug reactions (Walters and Woessner, 2016). While the market growth of COX-2 inhibitors, introduced in recent years, has slowed down, concerns about their safety, particularly regarding cardiovascular side effects, are on the rise (Mukherjee et al., 2001). Furthermore, recent studies have shown that the clinical pain relief provided by commonly used doses of ampiroxicam is not substantialy different from that provided by the COX-2 inhibitor meloxicam (Aoki et al., 2006). Therefore, there is an urgent need to develop NSAIDs that are both safe and effective for use in clinical settings, while minimizing side effects. Recent efforts have primarily focused on reducing adverse gastrointestinal effects, with particular emphasis on prodrugs (Sehajpal et al., 2018; Gupta et al., 2021). As human health needs continue to evolve, products with considerable toxicity and poor efficacy are being phased out, making way for those with proven efficacy, minimal adverse reactions, and good tolerance. In the clinical setting, the replacement of piroxicam with ampiroxicam has become an inevitable shift. With the continuous exploration of new applications, precursor drugs similar to oxicams are strongly believed to experience substantial growth in the future; thus potentially emerging as pivotal players in the fields of fever reduction, pain relief, and anti-inflammatory medications. A thorough understanding of ampiroxicam’s PK characteristics is critical for optimizing its dosing regimen, thereby enhancing its therapeutic efficacy while minimizing adverse effects. Given the significant gastrointestinal and cardiovascular side effects associated with traditional NSAIDs, a detailed PK analysis of ampiroxicam is particularly important. This analysis will provide insights into its safety and efficacy, positioning ampiroxicam tablets as a potentially safer alternative in the NSAID class. By characterizing the ADME properties of ampiroxicam in tablet form, this study aims to inform clinical decision-making and guide the development of dosing strategies that maximize patient benefit and minimize the risk of adverse reactions. The entire research process is illustrated in Figure 1.

## Materials and methods

### Formulations and subject selection

Ampiroxicam tablets were supplied by Shenyang Everbright Pharmaceutical Co., Ltd. Each tablet contains 13.5 mg of the active ingredient, with a batch number of 20,230,616 and a shelf life of 2 years. This study included 40 participants, with an equal distribution of 20 males and 20 females, aged between 18 and 40 years. Notably, participants within the same batch did not exhibit an age difference exceeding 10 years, and their Body Mass Index fell within the range of 19–25. All subjects underwent standard blood and urine tests as well as assessments of liver and kidney function and electrocardiograms, all of which returned normal results. Written informed consent was obtained from each participant in strict adherence to the Declaration of Helsinki and Good Clinical Practice guidelines. Prior to providing consent, the participants were provided with a comprehensive understanding of the properties of the drug, research objectives, potential risks, and their rights and obligations. Researchers recorded data based on each participant’s initial health status, ensuring accuracy, completeness, and timeliness on the “Case Report Form.” Monitoring throughout the study adhered to the study’s established protocols, confirming the accuracy and completeness of all “Case Report Forms.” In the event of any necessary modifications, the original records were clearly annotated, bearing the researcher’s signature and date of modification. Once the monitors were satisfied that the study’s typical charts, blood concentration data, and “Case Report Forms” were free from errors, they officially approved them. Subsequently, the data were transferred to a clinical data analysis team for an in-depth analysis.This study was conducted at the National Institute for Drug Clinical Experiments of the First Affiliated Hospital of the China Medical University.

### Chemical materials

The piroxicam reference standard (Supplementary Figure S1B) used in this study was of high purity (99.6%) and sourced from the National Institute for the Control of Pharmaceutical and Biological Products (Beijing, China). The meloxicam reference standard employed in this study had a purity of 99.7% and was obtained from the National Institute for the Control of Pharmaceutical and Biological Products (Beijing, China). Chromatographic analysis was performed using acetonitrile (Merck, Darmstadt, Germany) and phosphoric acid supplied by the American Tedia Company, Inc., both of which met the stringent standards for chromatographic purity. The chemical reagents used included hydrochloric acid and triethylamine (both provided by the China National Pharmaceutical Group Chemical Reagent Co., Ltd.), they all met the superior reagent grade criteria. The study used Ultrapure water which was supplied by Millipore (Billerica, MA, United States). Blank plasma, which served as a control sample, was provided by the Liaoning Provincial Blood Centre.

### Dosing and administration

Prior to medication administration, subjects fasted for at least 10 h. The following morning, at 7:00 a.m., they ingested the experimental drug on an empty stomach, accompanied by 250 mL of lukewarm water. The product specification for each tablet was 13.5 mg. In the absence of well-established pharmacokinetic data for ampiroxicam oral tablets, the dosage strategy was determined by referencing the pharmacokinetic literature of piroxicam oral tablets (Rasetti-Escargueil and Grangé, 2005; Wang et al., 2000; Deroubaix et al., 1995). This approach also accounts for the need for precise dosing in practical applications and exploration of the linear relationship between the area under the curve (AUC) and dosage. For the single-dose regimen, the participants were categorized into three dosage groups: low-dose (13.5 mg), medium-dose (27 mg), and high-dose (54 mg). The multiple-dose regimen involved the daily administration of ampiroxicam tablets at 27 mg for a continuous duration of 10 days.

### Single-dose and multiple-dose pharmacokinetic study design

This study utilizes the DAS system to select the corresponding seed number for randomization. The single-dose clinical pharmacokinetic study involved 30 participants with an equal sex distribution, who were further categorized based on body weight and randomly assigned to three groups, each comprising 10 individuals. In this study, participants received single doses of 13.5, 27, and 54 mg, evenly distributed among low-, medium-, and high-dose groups, with 10 individuals in each category. The study protocol required the participants to fast overnight, and the subsequent morning, they took the medication on an empty stomach. Participants in the low-, medium-, and high-dose groups orally ingested one, two, and four tablets of the investigational drug, respectively, with 250 mL of lukewarm water. After dosing, the participants were instructed to remain upright for the first 2 h, adopt a semi-reclining position, avoid water intake, and abstain from food for the first 4 h. A standardized meal (low-fat diet) was provided to all the participants 4 h after dosing. Blood samples were collected at specific intervals based on a single-dose regimen. In the single-dose group, blood samples were collected before dosing (0 h) and at 0.5, 1, 2, 4, 6, 9, 12, 24, 48, 72, 120, 168, and 216 h after dosing.

This multiple-dose pharmacokinetic study involved 10 participants, equally divided between males and females, to explore the pharmacokinetics of ampiroxicam at a dosage of 27 mg per administration. In the multiple-dose group, each of the 10 participants received two tablets of ampiroxicam, accompanied by 250 mL of lukewarm water, once daily for a consecutive 10 day period. To evaluate the time required to reach a steady state and assess trough concentrations, blood samples were collected on days 7th, 8th, and 9th days just before dosing. The final dose was administered on the 10th day, and blood samples were collected before dosing (0 h) and at various post-dose time points (0.5, 1, 2, 4, 6, 9, 12, 24, 48, 72, 120, 168, and 216 h).

Each blood sample comprised 4 mL of venous blood collected from the elbow, treated with heparin for anticoagulation, and centrifuged to isolate the plasma. The collected plasma samples were preserved at −40°C and later employed for the analysis of drug concentration in the blood. Throughout the study, the participants were accommodated in phase I patient rooms, instructed to avoid strenuous physical activity, and advised against prolonged bed rest. Additionally, they were instructed to refrain from consuming beverages containing caffeine, alcohol, tea, or coffee.

### Instrumentation and conditions

The analytical setup included a UPLC system (Waters Corporation, Milford, MA, United States), which comprised a high-pressure quadruple pump, an online degassing system, an automatic sampler, a column oven, and a TUV detector. Data were acquired and processed using an Empower chromatography workstation (Waters Corporation, Milford, MA, United States). The Milli-Q Gradient A10 ultrapure water system was provided by Millipore, Inc. (Billerica, MA, United States). The TGL-16C centrifuge was manufactured by Shanghai Anting Scientific Instrument Co., Ltd. (Shanghai, China), and the XW-80A Mini Vortex Mixer was sourced from the Huxi Instrument Factory (Shanghai, China). Chromatographic conditions were maintained as follows: an Acquity UPLC HSS T3 column (2.1 mm × 100 mm, 1.8 μm) was held at a constant temperature of 35°C. The mobile phase consisted of acetonitrile and water (with 0.21% triethylamine), with the pH adjusted to three using phosphoric acid in a ratio of 47:53. The flow rate was set at 0.6 mL/min, with an injection volume of 10 μL. The detection was performed at a wavelength of 360 nm. The instruments and chromatographic conditions were carefully chosen to ensure the precision and reliability of the analytical process.

### Plasma sample processing

A precise volume of 100 μL of plasma was carefully transferred into a 1.5 mL centrifuge tube. Subsequently, 10 μL of the internal standard solution of meloxicam (at a concentration of 18.00 μg/mL) was accurately added. The mixture was vortexed for 30 s. Following this, 200 μL of acetonitrile (containing 0.1 mol/L hydrochloric acid) was carefully added, and the sample was vortexed for 2 min. The centrifuge was set at 14,000 rpm for 10 min, and the supernatant was collected. A 10 μL supernatant was used for injection into the analysis system.

## Method specificity

Chromatographic analysis involved preparation of distinct sample sets: blank plasma (Supplementary Figure S2A), drug-spiked plasma (10.0 μg/mL, Supplementary Figure S2B), and piroxicam standard solution (Supplementary Figure S2C) were processed following “Plasma sample processing” guidelines. Additionally, plasma from a subject receiving 54 mg of piroxicam was analyzed (Supplementary Figure S2D). These steps ensured accurate and interference-free determination of piroxicam and the internal standard meloxicam in plasma samples. Piroxicam exhibited a retention time of approximately 1.5 min, and the internal standard meloxicam was detected at around 2.2 min, providing reliable chromatograms for precise drug detection.

### Calibration curve and LLOQ

In this procedure, varying concentrations of piroxicam standard solution (10 μL) were pipetted into empty centrifuge tubes. Following the “Plasma sample processing” steps, samples were prepared for analysis, and a standard curve for piroxicam was established. The curve demonstrated a strong linear relationship within the range of 0.02–12.00 μg/mL, covering expected human blood concentrations. The quantification limit was determined as 0.02 μg/mL. Systematic errors were minimized by analyzing a sample with a known concentration (0.02 μg/mL) alongside other samples. UPLC-UV accurately measured piroxicam down to 0.02 μg/mL. Standard curves and quality control samples further ensured precision across batches. Detailed results are in Supplementary Tables S1–S8, and Supplementary Figure S3 displays the standard curves.

### Precision and accuracy

The quality control samples were prepared in empty centrifuge tubes. Each tube received 10 μL of piroxicam standard solution at varying concentrations, followed by the “Plasma sample processing” steps. This process yielded plasma samples with drug concentrations set at 0.04, 1.00, and 10.0 μg/mL, representing low, medium, and high levels, respectively. Over the course of several consecutive days, three separate analytical batches were prepared and analyzed. Within each batch, six samples were examined at each concentration. Detailed results are shown in Supplementary Table S9. These precisely crafted quality control samples were assessed to gauge the consistency and reliability of the method for varying concentrations on different days.

### Extraction recovery

In this process, empty centrifuge tubes were used as containers. Each tube received 10 μL piroxicam standard solution at varying concentrations, followed by the “Plasma sample processing” guidelines. Samples at low, medium, and high concentrations were created, each analyzed six times. Drug [As(H)] and internal standard peak areas [Ai(H)] were determined. Different concentrations of piroxicam and internal standard solutions were accurately diluted and analyzed, obtaining drug [As(D)] and internal standard peak areas [Ai(D)]. Extraction recovery, calculated as As(H)/As(D) × 100% for the drug and Ai(H)/Ai(D) × 100% for the Internal Standard, consistently fell within 90.81%–109.86%. Internal standard recovery ranged from 96.35% to 105.73%, affirming the robustness of the extraction recovery process across concentration levels, ensuring accurate and precise analysis. The comprehensive results are presented in Supplementary Table S10.

### Stability assessment of piroxicam standard solutions and plasma samples

Experiments assessed the stability of piroxicam standard solutions using procedures. Solutions were prepared, analyzed, and stored under various conditions. Results consistently demonstrated peak area ratios with RSD values below 10%, confirming the stability of piroxicam standard solutions. The weighing, preparation, and analysis procedures ensured accurate and reliable outcomes. Detailed (Supplementary Tables S11, S12) provide comprehensive data on peak areas and RSD values, attesting to the robustness of the methodology under diverse conditions, including storage at 4°C for 20 days.

Piroxicam plasma stability was evaluated at different concentrations. Samples underwent various conditions, including room temperature storage, freeze-thaw cycles, and long-term freezing. RSD values consistently below 10% in triplicate analyses indicated piroxicam’s robust stability. The method, tested under ambient and extreme conditions, demonstrates reliability for assessing plasma stability, critical for accurate pharmacokinetic investigations. Results, detailed in Supplementary Table S13, affirm piroxicam’s stability under diverse scenarios, supporting its suitability for analytical precision in plasma samples.

### Data processing

Pharmacokinetic parameters were computed and subjected to statistical analysis using DAS2.1.1 and SPSS 11.0 software. The primary focus was descriptive statistics, with inferential statistics serving as references. Numerical data are presented as mean ± standard deviation (Mean ± SD). To determine whether the changes in various parameters before and after administration or between dosage groups were statistically significant, a significance level of *P* < 0.05 was employed.

## Results

### Determination of plasma samples and analysis of results

**FIGURE 2:**
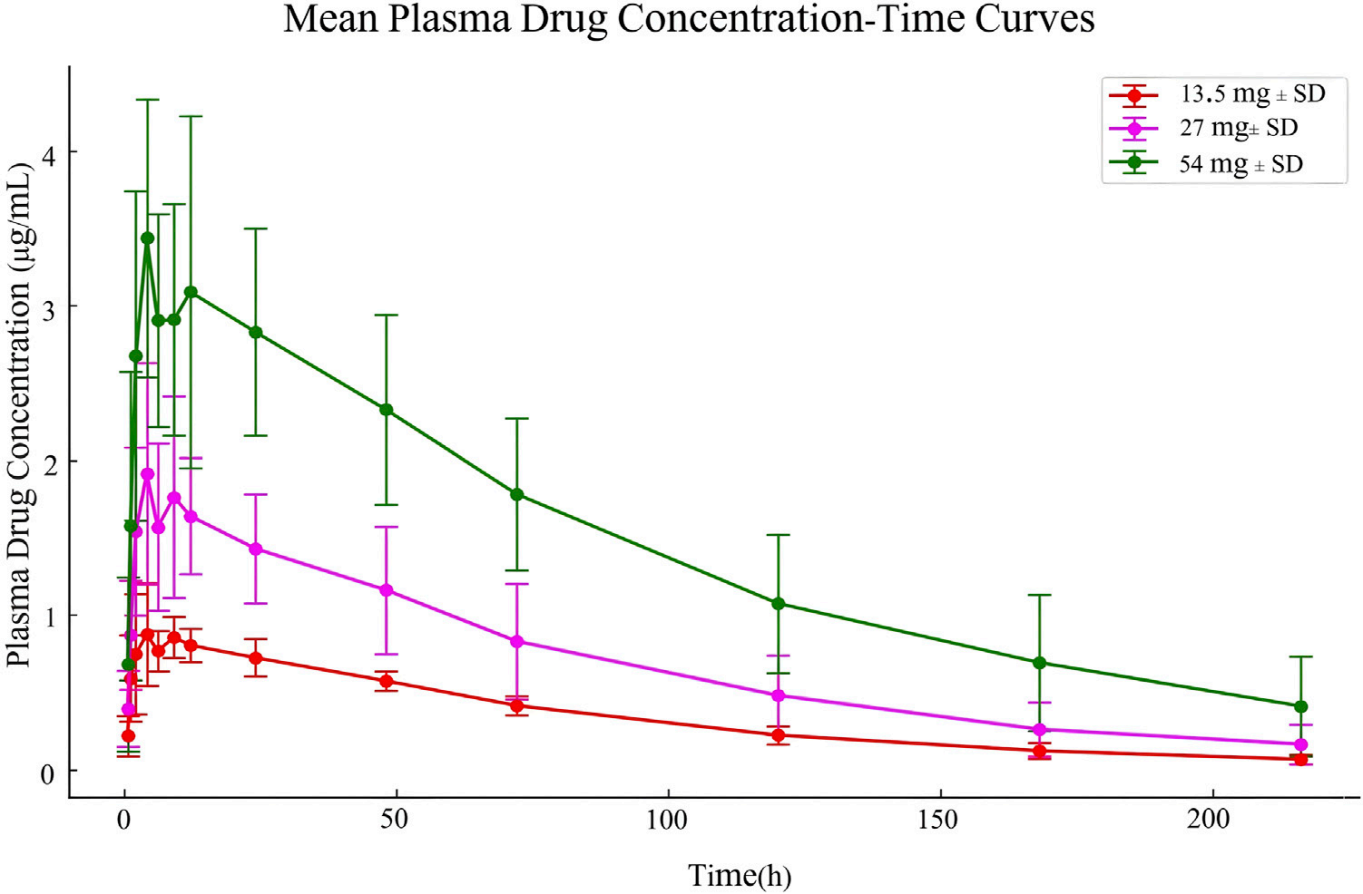
Mean plasma drug concentration-time curves in Study Participants Following Single Dose Oral Administration of 13.5 mg, 27 mg, and 54 mg Ampiroxicam Tablets.

**FIGURE 3:**
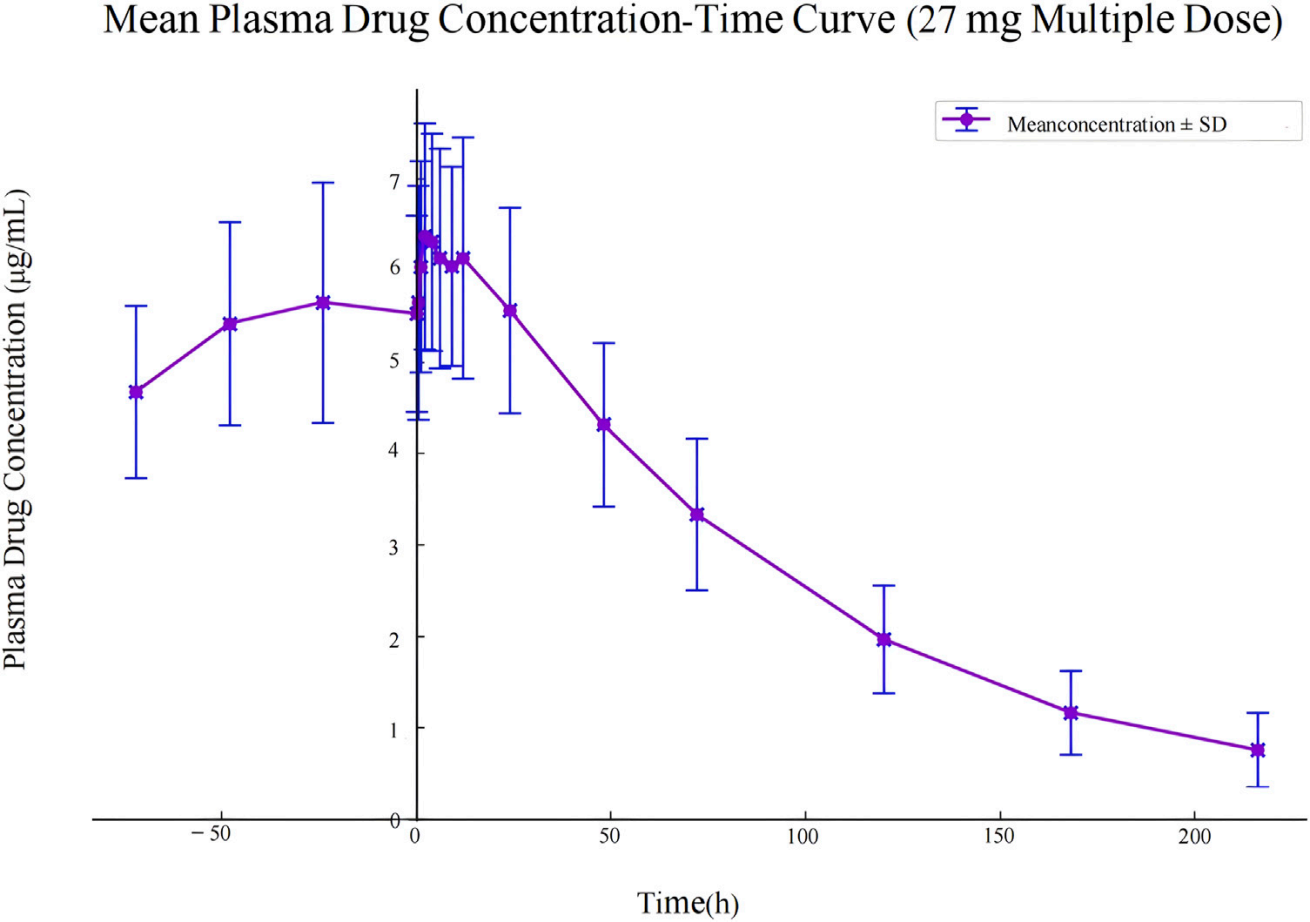
Mean plasma drug concentration-time curve in study participants following multiple dose administration of 27 mg ampiroxicam.

The pharmacokinetic data obtained from 30 participants after a single dose of ampiroxicam tablets are detailed in Supplementary Tables S14–S16, as well as in Figure 2. Data from 10 participants who received multiple doses of ampiroxicam tablets are shown in Supplementary Table S17. Furthermore, Figure 3 displays the average concentration-time profiles of piroxicam in the plasma of 40 healthy subjects following both a single dose and multiple doses. These findings provide a comprehensive understanding of the changes in drug concentration over time in response to ampiroxicam administration in the studied individuals.

### Presentation and analysis methods for single-dose and multiple-dose administration trials

For single-dose administration, pharmacokinetic analysis was conducted using compartmental and non-compartmental models. Individual pharmacokinetic parameters, including t_max_, C_max_, AUC, V, t_1/2_, and CL, were calculated for each participant. Mean and standard deviation were computed for each parameter.

For multiple-dose administration, compartmental and non-compartmental models were employed. This time, pharmacokinetic parameters were calculated after the last dose, including t_max_, V, t_1/2_, CL, AUC_ss_, C_av_, DF, C_max_, and C_min_. Mean and standard deviation were calculated for each parameter. t_max_ and C_max_ were determined from observed values, while AUC_0–216_ and AUC_0–∞_ were computed using the trapezoidal method. CLz (clearance) was calculated as D/AUC, where D represents the administered dose. Volume of distribution (Vz) was derived from CLz/ke and mean residence time (MRT) was calculated as area under the first moment curve divided by the AUC.

### Pharmacokinetic calculations

Analysis of the pharmacokinetic parameters was conducted utilizing DAS2.1.1 software, which revealed the conformity of these parameters to a two-compartment model. The pharmacokinetic parameters of 10 participants in the low-dose group (13.5 mg) after a single administration are shown in Supplementary Tables S18, S19. The medium-dose group (27 mg) also received a single dose, and the respective pharmacokinetic parameters for the 10 participants are presented in Supplementary Tables S20, S21. Likewise, the high-dose group (54 mg) received a single dose. The pharmacokinetic parameters of these 10 participants are detailed in Supplementary Tables S22, S23. The pharmacokinetic parameters of the multiple-dose group (27 mg), which consisted of 10 participants, after repeated dosing, are summarized in Supplementary Tables S24, S25. These findings offer comprehensive insights into the pharmacokinetic profiles of varying dosage groups.

### Correlation analysis of pharmacokinetic parameters

Using DAS2.1.1 statistical software, a correlation analysis was conducted between dosage and statistical moment parameters, which include C_max_, AUC_0–216_, and AUC_0–∞_. Subjects receiving single low (13.5 mg), medium (27 mg), and high (54 mg) doses of the medication displayed a notable linear relationship between AUC_0-t_, AUC_0–∞_, C_max_ (represented as Y), and the administered dosage (represented as X), as illustrated in Table 26–28. The correlations were strong: Y = −8.669 + 6.031X (*R* = 0.839), Y = −19.297 + 7.085X (*R* = 0.783), and Y = 0.153 + 0.064X (*R* = 0.822) (where Y represents the lnAUC and X represents the dosage administered). The dose-response relationships between the high, medium, and low doses are detailed in Supplementary Tables S26–S28, with R values of 0.839, 0.783, and 0.822, respectively (all *R* ^2^ values exceeding 0.500). This indicates a strong positive correlation between dosage and AUC_0–216_, AUC_0–∞_, and C_max_.

### Comparison of major pharmacokinetic parameters in different dosage groups

Pharmacokinetic parameters following the oral administration of 13.5, 27, and 54 mg of ampiroxicam to 30 healthy participants were subjected to analysis of variance, and the results are presented in Supplementary Table S29. Using SPSS 11.0, normal distribution tests were conducted for the within-group statistical moment parameters, including t_1/2_, CL, V, AUC_0–216_, AUC_0–∞_, MRT_0–216_, and C_max_. Additionally, tests for the homogeneity of variance between the dosage groups were performed. Subsequently, a one-way analysis of variance was performed to compare the various dosage groups. For the analysis of AUC_0–216_, AUC_0–∞_, and C_max_, normalization was initially applied by dividing these values by the respective doses: AUC_0–216_/13.5, AUC_0–∞_/13.5, C_max_/13.5 for the 13.5 mg single-dose group; AUC_0–216_/27, AUC_0–∞_/27, C_max_/27 for the 27 mg single-dose group; and AUC_0–216_/54, AUC_0–∞_/54, C_max_/54 for the 54 mg single-dose group, followed by statistical analysis. The t_max_ was subjected to non-parametric testing for statistical analysis. The results of the variance analysis revealed that there were no statistically significant differences (*p* > 0.05) among the pharmacokinetic parameters, including t_1/2_, CL, V, MRT_0–216_, and the normalized AUC_0–216_, AUC_0–∞_, C_max_ for the 13.5 mg, 27 mg, and 54 mg dosage groups (See Tables 1, 2 and Supplementary Table S30). Non-parametric testing also indicated no statistically significant differences (*p* > 0.05) in tmax among the three dosage groups (Supplementary Table S31). Following multiple doses, blood trough concentrations (C_min_) measured on the 7th, 8th, and 9th days are presented in the following table, with the results of the variance analysis shown in Table 3. Table 3 also shows that there were no significant differences in the blood trough concentrations before medication on days 7th, 8th, and 9th days of continuous ampiroxicam administration (*P* > 0.05). This suggests that a steady state was achieved by the 7th day of continuous ampiroxicam administration.

**TABLE 1:** Analysis of variance (ANOVA) results for primary pharmacokinetic parameters following single oral administration of ampiroxicam at different dosages.

Dosage (mg)		AUC _0–216_ (mg·h/L)	AUC –_0–∞_ (mg·h/L)	t_1/2_ (h)	C_max_ (mg/L)	CL (L/h)	V (L)	MRT (h)
13.5	$\bar{x}$	74.562	80.484	54.723	0.989	0.171	13.202	64.963
	±s	9.508	12.711	11.914	0.180	0.026	1.545	6.544
27	$\bar{x}$	151.661	165.856	55.169	1.973	0.186	14.048	63.915
	±s	59.798	72.374	11.717	0.679	0.064	3.553	9.019
54	$\bar{x}$	317.844	365.324	65.068	3.595	0.169	14.469	70.250
	±s	101.220	152.317	20.140	1.140	0.061	1.993	8.366
	F	33.334	22.414	1.498	28.981	0.293	0.658	1.782
	P	<0.001	<0.001	0.242	<0.001	0.748	0.526	0.188

**TABLE 2:** Analysis of Variance (ANOVA) Results for Primary Pharmacokinetic Parameters and dose ratios Following Single Oral Administration of Ampiroxicam at Different Dosages.

Dosage (mg)		AUC _0–216_/dose (mg·h/L)	AUC _0–∞_/dose (mg·h/L)	C_max_/dose (mg/L)
13.5	$\bar{x}$	5.523	5.962	0.073
	±s	0.704	0.942	0.013
27	$\bar{x}$	5.617	6.143	0.073
	±s	2.215	2.680	0.025
54	$\bar{x}$	5.886	6.765	0.067
	±s	1.874	2.821	0.021
	F	0.119	0.332	0.353
	P	0.888	0.720	0.706

**TABLE 3:** Analysis of variance (ANOVA) results for trough concentrations (Cmin) following continuous oral administration of 27 mg ampiroxicam.

Dose of administration			27 mg
**C**_**min**_, _**av**_ ± s	Day 7	$\bar{x}$ ± s	4.677 ± 0.939
	Day 8	$\bar{x}$ ± s	5.424 ± 1.114
	Day 9	$\bar{x}$ ± s	5.655 ± 1.311
*P*-value			0.150

### Comparison of major pharmacokinetic parameters in different dosage groups for male and female participants

Healthy male and female participants who received single oral doses of 13.5, 27, and 54 mg of ampiroxicam underwent intergroup t-tests for pharmacokinetic parameters; the results are presented in Table 4. Table 4 indicates that following the single oral administration of 13.5, 27, and 54 mg of ampiroxicam, there were no statistically significant differences (*P* > 0.05) in various pharmacokinetic parameters, including AUC_0–216_, AUC_0–∞_, t_1/2_, C_max_, CL, V, and MRT, between male and female participants.

**TABLE 4:** *t*-test results for pharmacokinetic parameters in male and female subjects following single oral administration of ampiroxicam at different dosages.

Dosage (mg)	Gender		AUC _0–216_		AUC –_0–∞_	t_1/2_	C_max_	CL	V	MRT
	male	$\bar{x}$	73.778		79.342	52.768	0.997	0.173	12.833	65.626
		±s	6.660		9.970	14.140	0.220	0.020	2.060	8.180
13.5	female	$\bar{x}$	75.346		81.626	56.678	0.980	0.170	13.570	64.300
		±s	12.550		16.150	10.480	0.160	0.030	0.890	5.330
	*P*-value		0.811		0.795	0.633	0.895	0.894	0.484	0.769
	male	$\bar{x}$	127.495		135.511	51.692	1.630	0.210	15.618	62.686
		±s	33.440		35.850	3.010	0.460	0.050	3.740	3.510
27	female	$\bar{x}$	175.826		196.200	58.646	2.316	0.162	12.479	65.143
		±s	73.940		90.540	16.420	0.730	0.070	2.880	12.920
	*P*-value		0.220		0.201	0.379	0.113	0.257	0.175	0.692
	male	$\bar{x}$	313.129		368.944	67.347	3.348	0.177	15.065	72.018
		±s	126.070		192.390	25.510	1.570	0.080	2.310	7.030
54	female	$\bar{x}$	322.558		361.703	62.789	3.842	0.161	13.872	68.483
		±s	84.290		123.100	15.770	0.550	0.040	1.640	10.020
	*P*-value		0.893		0.945	0.743	0.525	0.706	0.374	0.536

For healthy male and female participants who received multiple oral doses of 27 mg ampiroxicam, the *t*-test results for pharmacokinetic parameters are presented in Table 5. Table 5 shows that, except for MRT, which exhibited statistical significance (*P* < 0.05), there were no statistically significant differences in other pharmacokinetic parameters, including C_max_, t_1/2_, CL, V, AUC_0–216_, AUC_0–∞_, between male and female participants following multiple oral doses of 27 mg of ampiroxicam. The observed difference in the MRT may be attributed to variations in body weight between healthy male and female participants. These results suggest that the pharmacokinetics of ampiroxicam in the human body do not exhibit sex differences.

**TABLE 5:** *t*-test results of pharmacokinetic parameters for male and female subjects after multiple oral doses of ampiroxicam.

Dosage (mg)	Gender		t_1/2_ (h		CL (L/h)	V (L)	C_max_ (mg/L)	C_min_ (mg/L)	C_av_ (mg/L)	DF (mg/L)	AUC_SS_ (mg·h/L)	AUC _0–t_ (mg·h/L)	AUC _0–∞_ (mg·h/L)	MRT (h)
27	male	$\bar{x}$ ± s	71.250 ± 20.230		0.040 ± 0.010	4.030 ± 0.790	6.110 ± 0.820	5.050 ± 0.770	5.490 ± 0.680	0.200 ± 0.030	131.800 ± 16.250	599.310 ± 102.370	693.070 ± 150.610	72.240 ± 3.600
	female	$\bar{x}$ ± s	53.440 ± s10.330		0.050 ± 0.020	3.360 ± 0.760	7.410 ± 1.350	6.010 ± 1.190	6.500 ± 1.390	0.220 ± 0.050	156.020 ± 33.360	607.770 ± 184.830	655.250 ± 217.480	63.700 ± 6.960
	*P*-value			0.118	0.589	0.207	0.103	0.167	0.183	0.365	0.183	0.931	0.757	0.041

### Tolerability

All 40 volunteers completed the trial. Both the single- and multiple-dose groups exhibited no adverse gastrointestinal effects such as nausea, stomach discomfort, or digestive issues throughout the experimental period. Additionally, no other symptoms such as dizziness, tinnitus, or headache were reported. There was only one instance (Participant 40) of a mild rash that occurred during the medication period, which was completely resolved by a follow-up physical examination after the conclusion of the study. Overall, this drug demonstrated a high level of safety with no substantial adverse reactions.

## Discussion

Due to the limited availability of publicly disclosed pharmacokinetic data for ampiroxicam (Olkkola et al., 1994; Ogiso et al., 1999), there is a lack of comprehensive information regarding methodologies and pharmacokinetics in healthy human subjects, and there is a notable dearth of literature on the pharmacokinetics of ampiroxicam oral tablets following ingestion in healthy individuals. This study presents the first detailed and comprehensive report on the pharmacokinetic profile of ampiroxicam oral tablets, both in single and multiple doses, in healthy subjects.

This study utilized a UPLC internal standard method for rapid and precise determination of piroxicam concentrations in plasma samples from healthy individuals. This approach not only ensures the accuracy of the measurements but also effectively mitigates the influence of endogenous substances on sample analysis. The method’s calibration curve displays a linear range spanning from 0.02 to 12.00 μg/mL, with a quantification limit of 0.02 μg/mL. The extraction recovery falls within a robust range of 90.81%–109.86%, while both intra- and inter-day precision remain comfortably below 15%. The method consistently achieved accuracy levels between 90.41% and 110.78%.

Several sample preparation techniques have been explored, including protein precipitation using methanol, various mixtures of hexane, dichloromethane, and isopropanol as the extraction solvent, ether-based extraction, and solid-phase extraction. However, these methods yield less-than-ideal results, leading to extended sample handling times, increased chromatographic peaks, and peak tailing. The selected sample preparation method involved direct protein precipitation in acetonitrile.

In contrast to previous methods for piroxicam and related drug analyses (Menshikova and Zakharova, 2021; Cho et al., 2022; Siddareddy et al., 2018; Salunkhe et al., 2019), this approach offers notable advantages, such as simplified sample preparation, shorter chromatographic retention times, and reduced reagent consumption. Furthermore, it fully aligns with the analytical requirements stipulated by both the NMPA, 2009; USFDA, 2001 for biological sample analyses.

In the subsequent analysis of pharmacokinetic parameters, a correlation analysis was performed between the pharmacokinetic parameters AUC_0–216_, AUC_0–∞_, and C_max_ and the administered dosage across the three dosage groups. The results indicated a dose-dependent increase in AUC_0–216_, AUC_0–∞_, and C_max_, highlighting a strong positive correlation between the administered dose and these parameters.

Furthermore, both variance and non-parametric analyses were applied to the key pharmacokinetic parameters within each dosage group. The findings revealed no statistically significant differences (*P* > 0.05) in the pharmacokinetic parameters t_1/2_, CL, MRT, V, as well as the normalized AUC_0–216_, AUC_0–∞_, and C_max_ among the 13.5, 27, and 54 mg dosage groups. The non-parametric test results also indicated no statistically significant differences (*P* > 0.05) in t_max_ among the three dosage groups.

We also analyzed the effect of sex on major pharmacokinetic data. Notably, apart from a statistically significant difference in MRT between sexes in the multi-dose group (*P* < 0.05), there were no statistically significant differences in other pharmacokinetic parameters across the different dosage groups. The pharmacokinetic parameters, including AUC_0–216_, AUC_0–∞_, C_max_, t_1/2_, CL, V, and MRT, exhibited no significant differences (*P* > 0.05) between male and female subjects. This suggests that the observed difference in MRT in the multidose group may be attributed to variations in the average body weight between male and female subjects.

Simultaneously, we conducted a detailed analysis of the pharmacokinetic properties of ampiroxicam as a prodrug of piroxicam. Our comparison revealed significant differences in pharmacokinetic parameters between ampiroxicam and piroxicam, particularly regarding half-life and C_max_. Our data indicate that the half-life of ampiroxicam ranges from 54.723 to 65.068 h, significantly longer than the approximately 50-hour half-life reported for piroxicam in the literature (Darragh et al., 1985; Wanwimolruk et al., 1991). This extended half-life may result from the metabolic conversion of ampiroxicam to piroxicam in the body, which likely slows the drug’s clearance rate and prolongs its duration of action. This finding is clinically significant, especially for treatments requiring sustained anti-inflammatory effects, as a longer t_1/2_ can reduce dosing frequency and improve patient compliance. Moreover, we observed that at the same dosage, C_max_ and AUC of ampiroxicam are higher than those of piroxicam. For instance, in our high-dose trial, the C_max_ of ampiroxicam reached 3.595 μg/mL, whereas piroxicam’s C_max_ typically ranges between 2.556 μg/mL and 2.658 μg/mL according to Al-Shakargi (2012). This suggests that ampiroxicam may offer higher bioavailability, aligning with its prodrug nature and potentially enhancing its efficacy. Based on these data, we propose that ampiroxicam may outperform piroxicam in certain clinical applications due to its pharmacokinetic advantages, particularly its longer half-life and higher bioavailability. Future research should further explore the clinical benefits of this prodrug, especially in managing chronic inflammatory diseases.

## Conclusion

As a class of drugs successful in antipyresis, analgesia, anti-inflammation, antirheumatic, anticoagulation, and even offering anticancer, anti-Alzheimer’s disease, ophthalmic disease treatment, and preterm birth prevention capabilities, NSAIDs have demonstrated substantial benefits in both their therapeutic effects and pharmacokinetics. Across diverse populations, NSAIDs have significantly enhanced quality of life while reducing the frequency and duration of fever, pain, and inflammation episodes. This study is dedicated to comprehensively evaluating the tolerability and pharmacokinetics of Ampiroxicam, administering single and multiple doses to healthy subjects and employing ultra-high-performance liquid chromatography for metabolite quantification, with the goal of providing clinical medication guidance for adult patients. The current literature on the pharmacokinetics of Ampiroxicam is scant, lacking sufficient information and research to detail the pharmacokinetics after single and multiple dosing, the body’s tolerance, and the impact of gender on medication effects. Given the limited data available, further research is required to understand Ampiroxicam’s adverse clinical reactions and its pharmacokinetics.

On another note, this study has successfully introduced a swift and effective analytical technique for detecting oxicams in human plasma. This methodology has demonstrated potential to improve the accuracy and safety of drug delivery in clinical trials. The findings indicate that its implementation could enhance clinical medication practices, benefiting healthcare professionals and patients by supporting more effective evidence-based dosaying strategies.

## Data Availability

The original contributions presented in the study are included in the article/Supplementary Material, further inquiries can be directed to the corresponding author.
