# Supplementary material for: Exploring the pharmacokinetics and tolerability of cyclooxygenase inhibitor ampiroxicam: a phase I study on single and multiple oral doses
Source: Front Pharmacol. 2024 Jun 21;15:1429971. doi: 10.3389/fphar.2024.1429971 (PMC11224448; doi:10.3389/fphar.2024.1429971)
Supplement: Supplementary file 1 [file Table1.docx]

**Table S1**

Peak Area Ratio and Curve Equation of Methodological Standard Curve on the First Day

| Concentration (μg/ml）  Peak Area Ratio | 0.02 | 0.05 | 0.10 | 0.50 | 2.50 | 5.00 | 12.00 |
| --- | --- | --- | --- | --- | --- | --- | --- |
| F1 | 0.0142 | 0.0350 | 0.0750 | 0.3413 | 1.7624 | 3.7605 | 9.1391 |
| F2 | 0.0158 | 0.0379 | 0.0756 | 0.3821 | 1.8424 | 3.6643 | 8.6620 |
| F3 | 0.0149 | 0.0369 | 0.0711 | 0.3366 | 1.7705 | 3.4443 | 7.4893 |
| Mean | 0.0150 | 0.0366 | 0.0739 | 0.3533 | 1.7918 | 3.6231 | 8.4301 |
| Average Standard Curve Equation | Y=0.715X＋0.001 | | | | | | |
| R^2^ | 1.000 | | | | | | |

**Table S2**

Peak Area Ratio and Curve Equation of Methodological Standard Curve on the Second Day

| Concentration (μg/ml）  Peak Area Ratio | 0.02 | 0.05 | 0.10 | 0.50 | 2.50 | 5.00 | 12.00 |
| --- | --- | --- | --- | --- | --- | --- | --- |
| F1 | 0.0160 | 0.0396 | 0.0732 | 0.3908 | 1.7936 | 3.5110 | 9.1933 |
| F2 | 0.0150 | 0.0353 | 0.0759 | 0.3830 | 1.9319 | 3.7874 | 8.4824 |
| F3 | 0.0138 | 0.0366 | 0.0739 | 0.3533 | 1.8134 | 3.6161 | 8.1670 |
| Mean | 0.0149 | 0.0371 | 0.0743 | 0.3757 | 1.8463 | 3.6382 | 8.6142 |
| Average Standard Curve Equation | Y= 0.735X＋0.0003 | | | | | | |
| R^2^ | 1.000 | | | | | | |

**Table S3**

Peak Area Ratios and Curve Equations of the Methodological Standard Curves on the Third Day

| Concentration(μg/ml）  Peak Area Ratio | 0.02 | 0.05 | 0.10 | 0.50 | 2.50 | 5.00 | 12.00 |
| --- | --- | --- | --- | --- | --- | --- | --- |
| F1 | 0.0169 | 0.0388 | 0.0781 | 0.3742 | 1.8908 | 3.6876 | 8.6923 |
| F2 | 0.0152 | 0.0376 | 0.0703 | 0.3762 | 1.8235 | 3.6464 | 8.2609 |
| F3 | 0.0172 | 0.0391 | 0.0724 | 0.3807 | 1.9076 | 3.7663 | 8.2783 |
| Mean | 0.0165 | 0.0385 | 0.0736 | 0.3770 | 1.8739 | 3.7001 | 8.4105 |
| Average Standard Curve Equation | Y =0.731X＋0.002 | | | | | | |
| R^2^ | 1.000 | | | | | | |

**Table S4**

Precision and Accuracy of Quantification of LLOQ in Piroxicam Plasma Samples (n = 5)

| Concentration (μg/ml） | Measured concentration（μg/ml） | Accuracy（％） | Mean | SD | RSD（%） |
| --- | --- | --- | --- | --- | --- |
| 0.02 | 0.019 | 93.98 |  |  |  |
|  | 0.019 | 95.67 |  |  |  |
|  | 0.019 | 93.85 | 0.0195 | 0.001 | 4.99 |
|  | 0.021 | 105.47 |  |  |  |
|  | 0.020 | 99.07 |  |  |  |

**Table S5**

Peak Area Ratios and Equations of the Piroxicam Calibration Curve

| Concentration (μg/ml） | F1 | F2 | F3 |
| --- | --- | --- | --- |
| 0.02 | 0.0174 | 0.0231 | 0.014 |
| 0.05 | 0.0370 | 0.0445 | 0.038 |
| 0.10 | 0.0738 | 0.0766 | 0.074 |
| 0.50 | 0.3607 | 0.3432 | 0.374 |
| 2.50 | 1.8382 | 1.7632 | 1.954 |
| 5.00 | 3.6938 | 3.3218 | 3.604 |
| 12.00 | 9.0820 | 8.2944 | 9.399 |
| Standard Curve Equation | Y=0.728X+0.002 | Y=0.680 X +0.010 | Y=0.757X-0.001 |
| R | 0.999 | 1.000 | 0.999 |

**Table S6**

Peak Area Ratios and Equations of the Piroxicam Calibration Curve

| Concentration (μg/ml） | F4 | F5 | F6 |
| --- | --- | --- | --- |
| 0.02 | 0.0165 | 0.014 | 0.0154 |
| 0.05 | 0.0398 | 0.033 | 0.0344 |
| 0.10 | 0.0730 | 0.064 | 0.0707 |
| 0.50 | 0.3682 | 0.326 | 0.3533 |
| 2.50 | 1.8381 | 1.802 | 1.7999 |
| 5.00 | 3.6021 | 3.138 | 3.4787 |
| 12.00 | 8.8110 | 7.715 | 8.1251 |
| Standard Curve Equation | Y=0.729X+0.002 | Y=0.652X+0.001 | Y=0.694X+0.001 |
| R | 1.000 | 0.999 | 1.000 |

**Table S7**

Peak Area Ratios and Equations of the Piroxicam Calibration Curve

| Concentration (μg/ml） | F7 | F8 | F9 | F10 |
| --- | --- | --- | --- | --- |
| 0.02 | 0.0182 | 0.0196 | 0.0171 | 0.0183 |
| 0.05 | 0.0385 | 0.0403 | 0.0392 | 0.0396 |
| 0.10 | 0.0708 | 0.0800 | 0.0743 | 0.0802 |
| 0.50 | 0.3672 | 0.3411 | 0.3712 | 0.3725 |
| 2.50 | 1.8131 | 1.7089 | 1.8396 | 1.8938 |
| 5.00 | 3.4371 | 3.2557 | 3.5959 | 3.6060 |
| 12.00 | 8.1018 | 8.0781 | 9.4197 | 9.5760 |
| Standard Curve Equation | Y=0.694X+0.004 | Y=0.676X+0.006 | Y=0.739X+0.002 | Y=0.752X+0.003 |
| R | 0.999 | 0.999 | 0.999 | 0.999 |

**Table S8**

Precision and Accuracy of Piroxicam Quality Control Samples

| No. | 0.04 μg/ml | | 1.00 μg/ml | | 10.0 μg/ml | |
| --- | --- | --- | --- | --- | --- | --- |
| 1 | 0.0420 | 105.06 | 1.0045 | 100.45 | 10.0158 | 100.16 |
| 2 | 0.0385 | 96.35 | 0.9822 | 98.22 | 9.9172 | 99.17 |
| 3 | 0.0396 | 99.07 | 1.0195 | 101.95 | 9.7606 | 97.61 |
| 4 | 0.0388 | 96.92 | 1.0514 | 105.14 | 10.7705 | 107.71 |
| 5 | 0.0394 | 98.54 | 1.0748 | 107.48 | 10.7929 | 107.93 |
| 6 | 0.0393 | 98.31 | 1.0753 | 107.53 | 10.9599 | 109.60 |
| 7 | 0.0403 | 100.85 | 0.9471 | 94.71 | 9.6001 | 96.00 |
| 8 | 0.0389 | 97.18 | 0.9802 | 98.02 | 9.7702 | 97.70 |
| 9 | 0.0387 | 96.70 | 0.9892 | 98.92 | 9.7600 | 97.60 |
| 10 | 0.0416 | 104.07 | 1.0012 | 100.12 | 9.6700 | 96.70 |
| 11 | 0.0376 | 93.99 | 1.0180 | 101.80 | 9.7651 | 97.65 |
| 12 | 0.0402 | 100.55 | 0.9829 | 98.29 | 9.9085 | 99.08 |
| 13 | 0.0411 | 102.75 | 1.0431 | 104.31 | 10.8471 | 108.47 |
| 14 | 0.0385 | 96.34 | 1.0314 | 103.14 | 10.3076 | 103.08 |
| 15 | 0.0354 | 88.48 | 0.9130 | 91.30 | 9.3817 | 93.82 |
| 16 | 0.0386 | 96.49 | 0.9911 | 99.11 | 9.6833 | 96.83 |
| 17 | 0.0355 | 88.77 | 1.0098 | 100.98 | 9.9408 | 99.41 |
| 18 | 0.0370 | 92.45 | 0.9672 | 96.72 | 10.1401 | 101.40 |
| 19 | 0.0353 | 88.16 | 1.0116 | 101.16 | 9.3212 | 93.21 |
| 20 | 0.0367 | 91.78 | 0.9923 | 99.23 | 8.7016 | 87.02 |
| 21 | 0.0400 | 100.05 | 1.0112 | 101.12 | 10.0940 | 100.94 |
| 22 | 0.0412 | 103.10 | 1.0210 | 102.10 | 9.7802 | 97.80 |
| SD | 0.0020 |  | 0.0398 |  | 0.5397 |  |
| Mean | 0.0388 |  | 1.0062 |  | 9.9495 |  |
| RSD(%) | 5.06 |  | 3.96 |  | 5.42 |  |

**Table S9**

Methodological Precision and Accuracy Experiments of Piroxicam Plasma Samples (n = 6)

|  | Piroxicam plasma concentrations（μg/ml） | | | | |  |
| --- | --- | --- | --- | --- | --- | --- |
| Determination date | 0.04 | Accuracy（％） | 1.00 | Accuracy（％） | 10.0 | Accuracy（％） |
| Day 1 | 0.0426 | 106.60 | 0.9507 | 95.07 | 10.4605 | 104.60 |
|  | 0.0411 | 102.75 | 1.0412 | 104.12 | 10.3341 | 103.34 |
|  | 0.0424 | 106.04 | 0.9915 | 99.15 | 9.9293 | 99.29 |
|  | 0.0433 | 108.28 | 1.0660 | 106.60 | 9.8404 | 98.40 |
|  | 0.0441 | 110.30 | 1.1078 | 110.78 | 9.5296 | 95.30 |
|  | 0.0438 | 109.60 | 1.0460 | 104.60 | 10.3857 | 103.86 |
| SD | 0.0011 |  | 0.0555 |  | 0.3704 |  |
| Mean | 0.0429 |  | 1.0339 |  | 10.0799 |  |
| RSD（%） | 2.57 |  | 5.37 |  | 3.67 |  |
|  | | | | | | |
| Day 2 | 0.0431 | 107.70 | 1.0450 | 104.50 | 10.3597 | 103.60 |
|  | 0.0392 | 97.94 | 1.0472 | 104.72 | 9.4237 | 94.24 |
|  | 0.0406 | 101.42 | 0.9911 | 99.11 | 9.5461 | 95.46 |
|  | 0.0408 | 101.96 | 1.0470 | 104.70 | 9.0691 | 90.69 |
|  | 0.0384 | 96.12 | 1.0665 | 106.65 | 10.4463 | 104.46 |
|  | 0.0403 | 100.87 | 1.0265 | 102.65 | 9.0413 | 90.41 |
| SD | 0.0016 |  | 0.0259 |  | 0.6177 |  |
| Mean | 0.0404 |  | 1.0372 |  | 9.6477 |  |
| RSD（%） | 3.94 |  | 2.50 |  | 6.40 |  |
|  | | | | | | |
| Day 3 | 0.0418 | 104.54 | 1.0018 | 100.18 | 9.9486 | 99.49 |
|  | 0.0403 | 100.87 | 0.9929 | 99.29 | 9.9966 | 99.97 |
|  | 0.0421 | 105.27 | 1.0340 | 103.40 | 9.2901 | 92.90 |
|  | 0.0378 | 94.49 | 1.0370 | 103.70 | 10.2919 | 102.92 |
|  | 0.0414 | 103.59 | 1.0496 | 104.96 | 9.9280 | 99.28 |
|  | 0.0419 | 104.69 | 1.0131 | 101.31 | 9.1123 | 91.12 |
| SD | 0.00164 |  | 0.02220 |  | 0.45675 |  |
| Mean | 0.0409 |  | 1.0214 |  | 9.7612 |  |
| RSD（%） | 4.01 |  | 2.17 |  | 4.68 |  |
|  | | | | | | |
| Inter-day SD | 0.0018 |  | 0.0360 |  | 0.4994 |  |
| Mean | 0.0414 |  | 1.0308 |  | 9.8296 |  |
| Inter-day RSD（%） | 4.28 |  | 3.49 |  | 5.08 |  |

**Table S10**

**Extraction Recovery Rates of Piroxicam in Plasma (n = 6)**

|  | Internal Standard Concentration（μg/ml） | Concentration of piroxicam in the plasma（μg/ml） | | |
| --- | --- | --- | --- | --- |
|  | 1.80 | 0.04 | 1.00 | 10.0 |
| rate of recovery (%) | 98.04 | 103.13 | 106.89 | 100.46 |
|  | 96.62 | 101.61 | 106.26 | 99.48 |
|  | 105.73 | 90.81 | 109.86 | 101.17 |
|  | 96.35 | 103.38 | 106.14 | 102.13 |
|  | 101.75 | 103.64 | 94.58 | 104.05 |
|  | 105.33 | 93.27 | 92.73 | 98.86 |
| SD | 4.25 | 5.73 | 7.19 | 1.89 |
| Mean | 100.63 | 99.31 | 102.74 | 101.03 |
| RSD（%） | 4.23 | 5.77 | 7.00 | 1.87 |

**Table S11**

Assessment of Room Temperature Stability of Piroxicam Standard Solution

| Concentration(μg/ml）  Peak Area Ratio  Time | 0.04（μg/ml） | 10.0（μg/ml） |
| --- | --- | --- |
| 0h | 0.0286 | 7.7422 |
|  | 0.0280 | 7.8481 |
|  | 0.0295 | 7.7644 |
| SD | 0.0007 | 0.0558 |
| Mean | 0.0287 | 7.7849 |
| RSD(%) | 2.60 | 0.72 |
| 8h | 0.0275 | 7.5628 |
|  | 0.0280 | 7.7611 |
|  | 0.0278 | 7.7314 |
| SD | 0.0003 | 0.1070 |
| Mean | 0.0278 | 7.6851 |
| RSD(%) | 0.97 | 1.39 |

**Table S12**

Assessment of Stability of Piroxicam Standard Solution Stored at 4°C for 20 Days

| Concentration(μg/ml）  Peak Area Ratio  Time | 0.04（μg/ml） | 10.0（μg/ml） |
| --- | --- | --- |
| 0h | 0.0340 | 6.4524 |
|  | 0.0339 | 6.6450 |
|  | 0.0327 | 6.5626 |
| SD | 0.0007 | 0.0966 |
| Mean | 0.0336 | 6.5533 |
| RSD(%) | 2.19 | 1.47 |
| 20d | 0.0334 | 6.3163 |
|  | 0.0360 | 7.3574 |
|  | 0.0336 | 6.6851 |
| SD | 0.0011 | 0.3626 |
| Mean | 0.0340 | 6.4524 |
| RSD(%) | 3.39 | 6.6450 |

**Table S13**

Assessment of Stability of Piroxicam Standard Solution Stored

| time | 0.04 | Accuracy（％） | 10.0 | Accuracy（％） |
| --- | --- | --- | --- | --- |
| 0h | 0.0426 | 106.60 | 10.4605 | 104.60 |
|  | 0.0411 | 102.75 | 10.3341 | 103.34 |
|  | 0.0424 | 106.04 | 9.9293 | 99.29 |
| SD | 0.0008 |  | 0.2775 |  |
| Mean | 0.0421 |  | 10.2413 |  |
| RSD(%) | 1.98 |  | 2.71 |  |
| Samples were treated for 24h at room temperature | 0.0416 | 103.94 | 10.4771 | 104.77 |
|  | 0.0388 | 97.09 | 9.0999 | 91.00 |
|  | 0.0383 | 95.64 | 10.1783 | 101.78 |
| SD | 0.0018 |  | 0.5215 |  |
| Mean | 0.0408 |  | 10.0799 |  |
| RSD(%) | 4.52 |  | 5.17 |  |
| Samples were treated at room temperature for 36h | 0.0428 | 106.97 | 10.0295 | 100.29 |
|  | 0.0380 | 95.02 | 10.2166 | 102.17 |
|  | 0.0418 | 104.54 | 9.9486 | 99.49 |
| SD | 0.0018 |  | 0.2184 |  |
| Mean | 0.0415 |  | 10.1531 |  |
| RSD(%) | 4.35 |  | 2.15 |  |
| Plasma at 8h after melting | 0.0394 | 98.51 | 9.0264 | 90.26 |
|  | 0.0405 | 101.21 | 9.2482 | 92.48 |
|  | 0.0395 | 98.81 | 10.0291 | 100.29 |
| SD | 0.0014 |  | 0.5805 |  |
| Mean | 0.0409 |  | 9.8379 |  |
| RSD(%) | 3.40 |  | 5.90 |  |
| Freeze and melt three times | 0.0411 | 102.79 | 9.3240 | 93.24 |
|  | 0.0415 | 103.71 | 9.7051 | 97.05 |
|  | 0.0406 | 101.47 | 9.4042 | 94.04 |
| SD | 0.0008 |  | 0.4710 |  |
| Mean | 0.0416 |  | 9.8595 |  |
| RSD(%) | 1.94 |  | 4.78 |  |
| Frozen for 20 days | 0.0407 | 101.73 | 9.3832 | 93.83 |
|  | 0.0401 | 100.36 | 9.4140 | 94.14 |
|  | 0.0404 | 101.09 | 9.4015 | 94.01 |
| SD | 0.0010 |  | 0.4934 |  |
| Mean | 0.0412 |  | 9.8204 |  |
| RSD(%) | 2.55 |  | 5.02 |  |
| Frozen for 40 days | 0.0416 | 104.06 | 9.2551 | 92.55 |
|  | 0.0410 | 102.49 | 9.3486 | 93.49 |
|  | 0.0402 | 100.56 | 9.0008 | 90.01 |
| SD | 0.0009 |  | 0.6067 |  |
| Mean | 0.0415 |  | 9.7214 |  |
| RSD(%) | 2.21 |  | 6.24 |  |

**Table S14**

Plasma Concentration-Time Data (μg/ml) after a single dose of 13.5mg Ampiroxicam in the 10 subjects

| T（h） | No.1 | No.2 | No.3 | No.4 | No.5 | No.6 | No.7 | No.8 | No.9 | No.10 | Mean | SD |
| --- | --- | --- | --- | --- | --- | --- | --- | --- | --- | --- | --- | --- |
| 0.5 | 0.2425 | 0.0483 | 0.4008 | 0.0272 | 0.2567 | 0.3223 | 0.1135 | 0.2721 | 0.3243 | 0.0000 | 0.223 | 0.131 |
| 1 | 0.7044 | 0.1306 | 0.8991 | 0.8604 | 0.4375 | 0.6922 | 0.1794 | 0.7151 | 0.7231 | 0.0000 | 0.594 | 0.28 |
| 2 | 1.2589 | 0.3459 | 1.0423 | 0.8339 | 0.6960 | 0.8912 | 0.3537 | 1.1581 | 0.8638 | 0.0516 | 0.75 | 0.389 |
| 4 | 1.1228 | 0.5477 | 1.1285 | 1.0530 | 0.7029 | 0.8579 | 0.9828 | 1.1865 | 1.0689 | 0.1254 | 0.878 | 0.334 |
| 6 | 0.7537 | 0.5617 | 1.0569 | 0.7288 | 0.7276 | 0.7474 | 0.8573 | 0.8372 | 0.7643 | 0.6636 | 0.77 | 0.131 |
| 9 | 0.8046 | 0.7583 | 0.9979 | 0.8248 | 0.7854 | 0.7241 | 0.8266 | 1.0784 | 1.0570 | 0.7276 | 0.858 | 0.134 |
| 12 | 0.9088 | 0.6458 | 0.8430 | 0.7443 | 0.7414 | 0.7228 | 0.7778 | 0.9112 | 0.9995 | 0.7721 | 0.807 | 0.107 |
| 24 | 0.7281 | 0.5921 | 0.8205 | 0.6037 | 0.7557 | 0.7177 | 0.5682 | 0.7508 | 0.9738 | 0.7451 | 0.726 | 0.12 |
| 48 | 0.4883 | 0.5290 | 0.6006 | 0.5985 | 0.5509 | 0.5509 | 0.5142 | 0.6057 | 0.6814 | 0.6530 | 0.577 | 0.061 |
| 72 | 0.3428 | 0.4822 | 0.4423 | 0.3493 | 0.4567 | 0.3341 | 0.4106 | 0.4455 | 0.5050 | 0.4145 | 0.418 | 0.06 |
| 120 | 0.3348 | 0.2669 | 0.2059 | 0.1517 | 0.2342 | 0.1787 | 0.1674 | 0.2008 | 0.2932 | 0.2401 | 0.227 | 0.058 |
| 168 | 0.0816 | 0.2028 | 0.1701 | 0.0657 | 0.1055 | 0.0576 | 0.1036 | 0.1334 | 0.1854 | 0.1531 | 0.126 | 0.051 |
| 216 | 0.0456 | 0.1316 | 0.0577 | 0.0403 | 0.0651 | 0.0393 | 0.0558 | 0.0781 | 0.1172 | 0.0775 | 0.071 | 0.032 |

Note: ND indicates values below the detection limit.

**Table S15**

Plasma concentration-time data after a single dose of 27mg Ampiroxicam in the 10 subjects (μg/mL)

| T（h） | No.1 | No.2 | No.3 | No.4 | No.5 | No.6 | No.7 | No.8 | No.9 | No.10 | Mean | SD |
| --- | --- | --- | --- | --- | --- | --- | --- | --- | --- | --- | --- | --- |
| 0.5 | 0.7684 | 0.2677 | 0.7948 | 0.4406 | 0.0486 | 0.0761 | 0.3963 | 0.4455 | 0.4059 | 0.3243 | 0.397 | 0.247 |
| 1 | 1.2938 | 0.8127 | 1.1716 | 0.9835 | 0.2453 | 0.3707 | 0.8600 | 1.2246 | 1.0690 | 0.7231 | 0.875 | 0.352 |
| 2 | 2.2411 | 1.1391 | 1.4783 | 1.4642 | 0.5440 | 1.3056 | 1.6706 | 2.4733 | 1.4139 | 1.7020 | 1.543 | 0.541 |
| 4 | 2.2991 | 1.4204 | 1.7465 | 1.6465 | 0.8079 | 2.3859 | 1.7889 | 3.5344 | 1.7901 | 1.7590 | 1.918 | 0.716 |
| 6 | 1.9041 | 1.3729 | 1.2550 | 1.4182 | 0.8943 | 1.8788 | 2.1108 | 2.4971 | 1.6068 | 0.7643 | 1.57 | 0.54 |
| 9 | 1.8747 | 1.3801 | 1.3862 | 1.6345 | 1.0105 | 2.3581 | 1.5427 | 3.3351 | 1.5747 | 1.5588 | 1.766 | 0.653 |
| 12 | 1.8568 | 1.3656 | 1.5591 | 1.4954 | 1.0366 | 2.0153 | 1.5360 | 2.4174 | 1.5675 | 1.5851 | 1.643 | 0.377 |
| 24 | 1.8156 | 1.2137 | 1.2396 | 1.1942 | 1.0036 | 1.7020 | 1.2039 | 2.1388 | 1.5083 | 1.2983 | 1.432 | 0.353 |
| 48 | 1.3584 | 0.8047 | 1.1604 | 0.9189 | 0.7515 | 1.5401 | 0.7884 | 2.0844 | 1.0761 | 1.1530 | 1.164 | 0.413 |
| 72 | 0.9894 | 0.5848 | 0.8239 | 0.5858 | 0.5555 | 1.0223 | 0.4106 | 1.7331 | 0.7663 | 0.8616 | 0.833 | 0.373 |
| 120 | 0.5054 | 0.2925 | 0.4763 | 0.3341 | 0.2406 | 0.6115 | 0.1911 | 1.0791 | 0.5129 | 0.5946 | 0.484 | 0.256 |
| 168 | 0.3009 | 0.1759 | 0.2665 | 0.1297 | 0.1295 | 0.3309 | 0.0748 | 0.6943 | 0.2363 | 0.3118 | 0.265 | 0.174 |
| 216 | 0.1397 | 0.0798 | 0.1343 | 0.0832 | 0.0811 | 0.1519 | ND | 0.4822 | 0.1301 | 0.2352 | 0.169 | 0.127 |

Note: ND indicates values below the detection limit.

**Table S16**

Plasma concentration-time data after a single dose of 54mg Ampiroxicam in the 10 subjects (μg/mL)

| T（h） | No.1 | No.2 | No.3 | No.4 | No.5 | No.6 | No.7 | No.8 | No.9 | No.10 | Mean | SD |
| --- | --- | --- | --- | --- | --- | --- | --- | --- | --- | --- | --- | --- |
| 0.5 | 1.1270 | 0.0000 | 0.7197 | 0.7761 | 0.0979 | 0.9578 | 0.0398 | 0.7011 | 1.7191 | 0.0279 | 0.685 | 0.564 |
| 1 | 2.3177 | 0.2412 | 3.0381 | 1.7008 | 0.2830 | 1.9981 | 0.2149 | 1.7301 | 2.3735 | 1.8942 | 1.579 | 0.997 |
| 2 | 3.5608 | 1.0569 | 3.3168 | 2.6454 | 1.3628 | 3.5736 | 1.3743 | 2.4980 | 3.7060 | 3.7065 | 2.68 | 1.064 |
| 4 | 3.8441 | 1.9439 | 4.3451 | 2.9372 | 2.1203 | 4.0034 | 3.3016 | 3.4128 | 4.6899 | 3.8037 | 3.44 | 0.897 |
| 6 | 3.1005 | 1.7115 | 3.5883 | 3.0261 | 2.0172 | 3.7260 | 2.4618 | 2.5781 | 3.4923 | 3.3819 | 2.908 | 0.69 |
| 9 | 2.8415 | 1.8391 | 4.0740 | 2.8358 | 2.0742 | 3.8664 | 2.1337 | 2.9018 | 3.4805 | 3.0867 | 2.913 | 0.752 |
| 12 | 2.8367 | 1.7582 | 5.8043 | 2.9062 | 2.0108 | 3.8025 | 2.3440 | 2.9879 | 3.5013 | 2.9823 | 3.093 | 1.138 |
| 24 | 2.7434 | 1.9054 | 3.8725 | 2.7730 | 1.9944 | 3.9643 | 2.7898 | 2.9846 | 2.7917 | 2.5094 | 2.833 | 0.672 |
| 48 | 2.0638 | 1.4786 | 3.2049 | 2.7323 | 1.9799 | 2.5814 | 2.0678 | 3.3489 | 2.0745 | 1.7695 | 2.33 | 0.614 |
| 72 | 1.7302 | 1.1461 | 2.5855 | 2.0423 | 1.3220 | 1.9189 | 1.5903 | 2.5442 | 1.4005 | 1.5675 | 1.785 | 0.49 |
| 120 | 0.9385 | 0.5438 | 1.8151 | 1.3761 | 0.8713 | 1.0849 | 0.9335 | 1.8019 | 0.7446 | 0.6678 | 1.078 | 0.448 |
| 168 | 0.4706 | 0.2869 | 1.5017 | 0.8195 | 0.4258 | 0.8272 | 0.4360 | 1.4036 | 0.4190 | 0.3605 | 0.695 | 0.438 |
| 216 | 0.2546 | 0.1177 | 1.0950 | 0.5657 | 0.2354 | 0.3807 | 0.2440 | 0.8481 | 0.2151 | 0.1982 | 0.415 | 0.323 |

Note: ND indicates values below the detection limit.

**Table S17**

Plasma Concentration-Time Data for 10 Subjects after Multiple Doses of 27mg Ampiroxicam

| T(h) | No.31 | No.32 | No.33 | No.34 | No.35 | No.36 | No.37 | No.38 | No.39 | No.40 | Mean | SD |
| --- | --- | --- | --- | --- | --- | --- | --- | --- | --- | --- | --- | --- |
| -72 | 4.5452 | 3.6059 | 4.4906 | 4.0780 | 4.1275 | 6.2519 | 3.3386 | 5.4532 | 5.5587 | 5.3222 | 4.677 | 0.939 |
| -48 | 5.3052 | 3.8373 | 4.7460 | 5.4699 | 5.0910 | 7.6547 | 4.0808 | 6.3009 | 6.1033 | 5.6460 | 5.424 | 1.114 |
| -24 | 5.6470 | 3.8688 | 5.1307 | 4.7915 | 5.2748 | 6.8901 | 3.7580 | 7.1051 | 6.9122 | 7.1697 | 5.655 | 1.311 |
| 0 | 5.6570 | 3.7623 | 4.9564 | 5.5725 | 5.2962 | 6.8414 | 3.9371 | 6.6773 | 6.4857 | 6.1180 | 5.53 | 1.073 |
| 0.5 | 5.9805 | 3.6824 | 4.9671 | 5.6200 | 5.1117 | 7.3665 | 4.1817 | 6.5808 | 7.6321 | 5.4329 | 5.656 | 1.277 |
| 1 | 6.4095 | 4.5378 | 5.3485 | 5.6653 | 5.4795 | 8.1961 | 4.7087 | 7.3919 | 6.6969 | 6.0253 | 6.046 | 1.155 |
| 2 | 6.3604 | 4.5707 | 5.3637 | 6.5154 | 6.6360 | 7.8882 | 4.5722 | 8.0650 | 6.4955 | 7.3452 | 6.381 | 1.235 |
| 4 | 6.4577 | 4.7664 | 5.8868 | 5.1730 | 5.4930 | 7.6016 | 5.2036 | 8.0963 | 7.6388 | 6.8506 | 6.317 | 1.187 |
| 6 | 5.9190 | 4.7039 | 5.4966 | 5.6347 | 5.9858 | 7.9737 | 4.2193 | 7.1862 | 7.2464 | 7.0057 | 6.137 | 1.198 |
| 9 | 5.8821 | 4.4751 | 5.3374 | 6.1223 | 5.6648 | 8.1292 | 4.9241 | 7.3104 | 6.6310 | 6.0403 | 6.052 | 1.09 |
| 12 | 6.7518 | 4.5794 | 5.8584 | 5.5333 | 5.5904 | 8.7736 | 4.1563 | 7.0487 | 6.7220 | 6.3881 | 6.14 | 1.319 |
| 24 | 5.8957 | 3.9324 | 5.2102 | 5.0433 | 5.8465 | 7.4401 | 3.8683 | 6.7237 | 6.1609 | 5.5606 | 5.568 | 1.123 |
| 48 | 5.1246 | 3.1890 | 4.3824 | 3.9886 | 4.0107 | 5.0292 | 2.6832 | 5.4927 | 5.0510 | 4.2354 | 4.319 | 0.897 |
| 72 | 4.0216 | 2.5758 | 3.4583 | 3.6343 | 3.4765 | 4.4555 | 1.5167 | 3.8621 | 3.4573 | 2.9127 | 3.337 | 0.83 |
| 120 | 2.5443 | 1.5099 | 2.1929 | 1.9503 | 2.3826 | 2.7687 | 0.7740 | 2.2556 | 1.8046 | 1.5349 | 1.972 | 0.59 |
| 168 | 1.6734 | 0.8097 | 1.3002 | 1.0260 | 1.2555 | 1.6735 | 0.3431 | 1.7178 | 1.1972 | 0.7023 | 1.17 | 0.458 |
| 216 | 1.4740 | 0.4749 | 0.7665 | 0.6586 | 1.3253 | 1.0200 | 0.2218 | 0.7075 | 0.5921 | 0.3560 | 0.76 | 0.404 |

Note: ND indicates values below the detection limit.

**Table S18**

Parameters of Compartment Model for 10 Subjects After a Single Dose of 13.5mg Ampiroxicam

| Subjects Number | Number of Compartment Models | t_1/2β_  (h) | V  (L) | CL  (L/h) | AUC_(0-t)_  (mg/L·h) | AUC_(0-∞)_  (mg/L·h) |
| --- | --- | --- | --- | --- | --- | --- |
| 1 (3) | 2 | 48.547 | 13.537 | 0.192 | 67.344 | 70.483 |
| 2 (4) | 2 | 69.315 | 17.832 | 0.149 | 76.058 | 90.715 |
| 3 (9) | 2 | 52.331 | 12.224 | 0.161 | 79.406 | 84.036 |
| 4 (12) | 2 | 47.373 | 14.717 | 0.219 | 59.573 | 61.692 |
| 5 (13) | 2 | 57.274 | 15.268 | 0.184 | 68.436 | 73.387 |
| 6 (17) | 2 | 45.836 | 14.765 | 0.223 | 58.36 | 60.654 |
| 7 (20) | 2 | 69.315 | 14.391 | 0.199 | 63.722 | 67.985 |
| 8 (23) | 2 | 69.315 | 12.344 | 0.160 | 77.862 | 84.188 |
| 9 (26) | 2 | 68.561 | 13.037 | 0.131 | 90.537 | 102.778 |
| 10 (28) | 2 | 57.725 | 13.208 | 0.171 | 72.519 | 79.081 |
| Mean |  | 58.559 | 14.132 | 0.179 | 71.382 | 77.500 |
| SD |  | 9.863 | 1.668 | 0.030 | 9.939 | 13.308 |

**Table S19**

Statistical Moment Parameters for a Single Dose of 13.5mg Ampiroxicam in 10 Subjects

| Subjects Number | t_1/2z_  (h) | T_max_  (h) | CL  (L/h) | V  (L) | C_max_  (mg/L) | AUC_(0-t)_  (mg/L·h) | AUC_(0-∞)_  (mg/L·h) | MRT_(0-t)_  (h) |
| --- | --- | --- | --- | --- | --- | --- | --- | --- |
| 1 ( 3) | 46.433 | 2 | 0.176 | 11.775 | 1.259 | 74.145 | 76.816 | 63.610 |
| 2 (4) | 76.829 | 9 | 0.148 | 16.400 | 0.758 | 76.676 | 91.262 | 79.064 |
| 3 (9) | 49.433 | 4 | 0.158 | 11.287 | 1.128 | 81.192 | 85.314 | 63.811 |
| 4 (12) | 39.816 | 4 | 0.209 | 11.981 | 1.053 | 63.100 | 64.738 | 56.731 |
| 5 (13) | 51.327 | 9 | 0.172 | 12.724 | 0.785 | 73.777 | 78.580 | 64.914 |
| 6 (17) | 44.093 | 2 | 0.204 | 12.985 | 0.891 | 63.646 | 66.152 | 56.785 |
| 7 (20) | 52.307 | 4 | 0.196 | 14.763 | 0.983 | 64.840 | 69.020 | 63.310 |
| 8 (23) | 55.027 | 4 | 0.159 | 12.651 | 1.186 | 78.951 | 84.735 | 62.719 |
| 9 (26) | 72.532 | 4 | 0.126 | 13.227 | 1.069 | 94.563 | 106.827 | 68.183 |
| 10 (28) | 59.431 | 12 | 0.166 | 14.224 | 0.772 | 74.728 | 81.396 | 70.501 |
| Mean | 54.723 | 5.4 | 0.171 | 13.202 | 0.989 | 74.562 | 80.484 | 64.963 |
| SD | 11.914 | 3.373 | 0.026 | 1.545 | 0.180 | 9.508 | 12.711 | 6.544 |

**Table S20**

Parameters of Compartment Model for 10 Subjects After a Single Dose of 27mg Ampiroxicam

| Subjects Number | Number of Compartment Models | t_1/2β_  (h) | V  (L) | CL  (L/h) | AUC_(0-t)_  (mg/L·h) | AUC_(0-∞)_  (mg/L·h) |
| --- | --- | --- | --- | --- | --- | --- |
| 11 (2) | 2 | 55.136 | 11.935 | 0.149 | 169.833 | 181.19 |
| 12 (5) | 2 | 52.122 | 17.577 | 0.232 | 110.041 | 116.506 |
| 13 (7) | 2 | 62.472 | 16.542 | 0.183 | 135.071 | 147.488 |
| 14 (11) | 2 | 48.923 | 15.825 | 0.222 | 116.111 | 121.877 |
| 15 (15) | 2 | 55.696 | 21.864 | 0.271 | 92.969 | 99.594 |
| 16 (16) | 2 | 54.229 | 10.756 | 0.137 | 184.774 | 197.662 |
| 17 (19) | 2 | 36.477 | 8.275 | 0.273 | 95.088 | 99.050 |
| 18 (22) | 2 | 69.315 | 9.195 | 0.078 | 292.236 | 344.46 |
| 19 (25) | 2 | 62.45 | 15.044 | 0.168 | 147.200 | 160.33 |
| 20 (29) | 2 | 69.315 | 17.21 | 0.146 | 156.777 | 184.312 |
| Mean |  | 56.614 | 14.422 | 0.186 | 150.01 | 165.247 |
| SD |  | 9.914 | 4.278 | 0.063 | 58.806 | 72.322 |

**Table S21**

Statistical Moment Parameters for a Single Dose of 27mg Ampiroxicam in 10 Subjects

| Subjects Number | t_1/2z_ (h) | T_max_ (h) | CL (L/h) | V (L) | C_max_ (mg/L) | AUC_(0-t)_ (mg/L·h) | AUC_(0-∞)_  (mg/L·h) | MRT_(0-t)_ (h) |
| --- | --- | --- | --- | --- | --- | --- | --- | --- |
| 11 (2) | 51.113 | 4 | 0.144 | 10.656 | 2.299 | 176.577 | 186.887 | 63.698 |
| 12 (5) | 50.277 | 4 | 0.234 | 16.958 | 1.420 | 109.717 | 115.510 | 60.885 |
| 13 (7) | 56.492 | 4 | 0.172 | 14.046 | 1.746 | 144.678 | 156.699 | 67.827 |
| 14 (11) | 48.425 | 4 | 0.223 | 15.612 | 1.646 | 115.011 | 120.852 | 58.370 |
| 15 (15) | 52.155 | 12 | 0.277 | 20.818 | 1.037 | 91.493 | 97.607 | 62.652 |
| 16 (16) | 54.076 | 4 | 0.134 | 10.473 | 2.386 | 187.219 | 201.172 | 66.417 |
| 17 (19) | 35.298 | 6 | 0.274 | 13.962 | 2.111 | 94.695 | 98.499 | 43.578 |
| 18 (22) | 75.655 | 4 | 0.079 | 8.600 | 3.534 | 293.908 | 342.761 | 76.599 |
| 19 (25) | 55.054 | 4 | 0.172 | 13.649 | 1.790 | 146.850 | 157.154 | 65.639 |
| 20 (29) | 73.146 | 4 | 0.149 | 15.709 | 1.759 | 156.458 | 181.414 | 73.480 |
| Mean | 55.169 | 5 | 0.186 | 14.048 | 1.973 | 151.661 | 165.856 | 63.915 |
| SD | 11.717 | 2.539 | 0.064 | 3.553 | 0.679 | 59.798 | 72.374 | 9.019 |

**Table S22**

Parameters of Compartment Model for 10 Subjects After a Single Dose of 54mg Ampiroxicam

| Subjects Number | Number of Compartment Models | t_1/2β_  (h) | V  (L) | CL  (L/h) | AUC_(0-t)_  (mg/L·h) | AUC_(0-∞)_  (mg/L·h) |
| --- | --- | --- | --- | --- | --- | --- |
| 21 (1) | 2 | 58.095 | 15.281 | 0.181 | 276.020 | 298.070 |
| 22 (6) | 2 | 52.672 | 23.530 | 0.312 | 164.485 | 173.292 |
| 23 (8) | 2 | 69.315 | 12.750 | 0.081 | 492.069 | 663.414 |
| 24 (10) | 2 | 69.315 | 16.513 | 0.134 | 338.357 | 401.595 |
| 25 (14) | 2 | 69.315 | 23.421 | 0.204 | 232.992 | 264.339 |
| 26 (18) | 2 | 62.923 | 12.522 | 0.137 | 357.669 | 393.601 |
| 27 (21) | 2 | 69.315 | 18.401 | 0.208 | 238.203 | 259.190 |
| 28 (24) | 2 | 69.315 | 16.661 | 0.101 | 407.485 | 535.217 |
| 29 (27) | 2 | 52.085 | 8.397 | 0.186 | 273.383 | 290.318 |
| 30 (30) | 2 | 65.152 | 14.652 | 0.203 | 250.547 | 266.107 |
| Mean |  | 63.750 | 16.213 | 0.175 | 303.121 | 354.514 |
| SD |  | 7.057 | 4.721 | 0.066 | 96.347 | 147.949 |

**Table S23**

Statistical Moment Parameters for a Single Dose of 54mg Ampiroxicam in 10 Subjects

| Subjects Number | t_1/2z_ (h) | T_max_ (h) | CL (L/h) | V (L) | C_max_ (mg/L) | AUC_(0-t)_ (mg/L·h) | AUC_(0-∞)_ (mg/L·h) | MRT_(0-t)_(h) |
| --- | --- | --- | --- | --- | --- | --- | --- | --- |
| 21 (1) | 55.751 | 4 | 0.175 | 14.084 | 3.844 | 287.829 | 308.462 | 65.976 |
| 22 (6) | 43.788 | 4 | 0.284 | 17.968 | 1.944 | 182.441 | 189.898 | 64.711 |
| 23 (8) | 108.654 | 12 | 0.08 | 12.477 | 5.804 | 506.217 | 678.594 | 81.074 |
| 24 (10) | 73.98 | 6 | 0.129 | 13.806 | 3.026 | 357.349 | 417.556 | 77.019 |
| 25 (14) | 54.561 | 4 | 0.216 | 16.992 | 2.120 | 231.810 | 250.211 | 71.309 |
| 26 (18) | 61.13 | 4 | 0.134 | 11.861 | 4.003 | 368.087 | 401.609 | 68.191 |
| 27 (21) | 54.273 | 4 | 0.188 | 14.76 | 3.302 | 267.666 | 286.519 | 68.007 |
| 28 (24) | 90.463 | 4 | 0.096 | 12.567 | 3.413 | 449.691 | 560.905 | 85.078 |
| 29 (27) | 53.304 | 4 | 0.186 | 14.274 | 4.690 | 274.479 | 290.996 | 60.189 |
| 30 (30) | 54.775 | 4 | 0.201 | 15.897 | 3.804 | 252.867 | 268.488 | 60.950 |
| Mean | 65.068 | 5 | 0.169 | 14.469 | 3.595 | 317.844 | 365.324 | 70.250 |
| SD | 20.140 | 2.539 | 0.061 | 1.993 | 1.140 | 101.220 | 152.317 | 8.366 |

**Table S24**

Parameters of Compartment Model for 10 Subjects After Multiple Doses of 27mg Ampiroxicam

| NO. | Number of Compartment Models | t_1/2β_  (h) | V  (L) | CL  (L/h) | AUC_(0-t)_  (mg/L·h) | AUC_(0-∞)_  (mg/L·h) |
| --- | --- | --- | --- | --- | --- | --- |
| 31 | 2 | 69.315 | 1.401 | 0.031 | 724.636 | 879.919 |
| 32 | 2 | 63.259 | 1.219 | 0.056 | 435.013 | 478.324 |
| 33 | 2 | 67.300 | 0.922 | 0.04 | 608.692 | 680.358 |
| 34 | 2 | 69.315 | 1.639 | 0.043 | 577.611 | 627.164 |
| 35 | 2 | 69.315 | 1.578 | 0.033 | 624.759 | 808.391 |
| 36 | 2 | 69.315 | 0.687 | 0.03 | 793.079 | 894.931 |
| 37 | 2 | 69.315 | 1.839 | 0.08 | 324.848 | 337.514 |
| 38 | 2 | 60.897 | 0.785 | 0.034 | 720.049 | 783.831 |
| 39 | 2 | 58.802 | 0.968 | 0.039 | 636.275 | 684.833 |
| 40 | 2 | 48.740 | 1.045 | 0.049 | 529.247 | 554.118 |
| Mean |  | 64.557 | 1.208 | 0.044 | 597.421 | 672.938 |
| SD |  | 6.824 | 0.391 | 0.015 | 140.475 | 179.369 |

**Table S25**

Statistical Moment Parameters for 10 Subjects After Multiple Doses of 27mg Ampiroxicam

| NO. | t_1/2z_ | T_max_ | CL | V | C_max_ | C_min_ | Cav | DF | AUC_SS_ | AUC_(0-t)_ | AUC_(0-∞)_ | MRT_(0-t)_ |
| --- | --- | --- | --- | --- | --- | --- | --- | --- | --- | --- | --- | --- |
|  | (h) | (h) | (L/h) | (L) | (mg/L) | (mg/L) | (mg/L) |  | (mg/L·h) | (mg/L·h) | (mg/L·h) | (h) |
| 31 | 74.744 | 12 | 0.032 | 3.459 | 6.752 | 5.657 | 6.255 | 0.175 | 150.124 | 726.464 | 841.861 | 76.531 |
| 32 | 61.046 | 4 | 0.055 | 4.889 | 4.766 | 3.762 | 4.404 | 0.228 | 105.699 | 444.890 | 486.527 | 68.325 |
| 33 | 63.29 | 4 | 0.040 | 3.622 | 5.887 | 4.956 | 5.521 | 0.169 | 132.507 | 610.769 | 680.838 | 72.326 |
| 34 | 52.603 | 2 | 0.044 | 3.305 | 6.515 | 5.573 | 5.533 | 0.170 | 132.784 | 578.533 | 620.051 | 69.019 |
| 35 | 104.552 | 2 | 0.032 | 4.872 | 6.636 | 5.296 | 5.746 | 0.233 | 137.895 | 635.894 | 836.083 | 74.997 |
| 36 | 67.724 | 12 | 0.030 | 2.928 | 8.774 | 6.841 | 8.056 | 0.240 | 193.341 | 801.430 | 901.270 | 70.510 |
| 37 | 40.411 | 4 | 0.080 | 4.676 | 5.204 | 3.937 | 4.316 | 0.293 | 103.574 | 327.933 | 336.686 | 53.969 |
| 38 | 56.839 | 4 | 0.034 | 2.807 | 8.096 | 6.677 | 7.162 | 0.198 | 171.897 | 731.150 | 788.988 | 69.468 |
| 39 | 55.072 | 4 | 0.039 | 3.130 | 7.639 | 6.486 | 6.703 | 0.172 | 160.870 | 638.786 | 685.561 | 65.030 |
| 40 | 47.176 | 2 | 0.048 | 3.260 | 7.345 | 6.118 | 6.266 | 0.196 | 150.393 | 539.554 | 563.750 | 59.506 |
| Mean | 62.346 | 5 | 0.043 | 3.695 | 6.761 | 5.530 | 5.996 | 0.207 | 143.908 | 603.540 | 674.162 | 67.968 |
| SD | 17.814 | 3.801 | 0.015 | 0.808 | 1.256 | 1.073 | 1.160 | 0.041 | 27.833 | 140.927 | 177.481 | 6.895 |

**Table S26**

Relationship Between Ampiroxicam Dosage and AUC_0-216_

|  | Parameter | SD | Confidence Interval | t-value | P-value |
| --- | --- | --- | --- | --- | --- |
| Intercept a | -8.669 | 27.315 | -64.716～47.377 | -0.317 | 0.753 |
| Slope b | 6.031 | 0.754 | 6.031～7.578 | 8.002 | <0.001 |
| R | 0.839 | 0.105 | 0.624～1.000 | 8.002 | <0.001 |
| Population mean of Y | 68.125 | 12.650 | 159.096～211.009 |  |  |

**Table S27**

Relationship Between Ampiroxicam Dosage and AUC_0-∞_

|  | Parameter | SD | Confidence Interval | t-value | P-value |
| --- | --- | --- | --- | --- | --- |
| Intercept a | -19.297 | 39.194 | -99.717～61.123 | -0.492 | 0.626 |
| Slope b | 7.085 | 1.081 | 7.085～9.304 | 6.551 | <0.001 |
| R | 0.783 | 0.120 | 0.538～1.000 | 6.551 | <0.001 |
| Population mean of Y | 97.751 | 18.152 | 171.025～245.514 |  |  |

**Table S28**

Relationship Between Ampiroxicam Dosage and C_max_

|  | Parameter | SD | Confidence Interval | t-value | P-value |
| --- | --- | --- | --- | --- | --- |
| Intercept a | 0.153 | 0.310 | -0.483～0.790 | 0.495 | 0.625 |
| Slope b | 0.064 | 0.009 | 0.064～0.082 | 7.504 | <0.001 |
| R | 0.822 | 0.110 | 0.597～1.000 | 7.504 | <0.001 |
| Population mean of Y | 0.774 | 0.144 | 1.922～2.512 |  |  |

**Table S29**

Primary Pharmacokinetic Parameters for Different Dosing Groups of Ampiroxicam Following Single Oral Administration Normality Assessment

| group |  | t_1/2_ | CL | V | MRT | AUC_0-216_ | AUC_0-∞_ | C_max_ |
| --- | --- | --- | --- | --- | --- | --- | --- | --- |
| 13.5mg | N | 10 | 10 | 10 | 10 | 10 | 10 | 10 |
|  | Kolmogorov-Smirnov Z | 0.600 | 0.410 | 0.612 | 0.642 | 0.528 | 0.481 | 0.538 |
|  | Asymp. Sig. (2-tailed) | 0.864 | 0.996 | 0.848 | 0.804 | 0.943 | 0.975 | 0.934 |
| 27mg | N | 10 | 10 | 10 | 10 | 10 | 10 | 10 |
|  | Kolmogorov-Smirnov Z | 0.807 | 0.584 | 0.491 | 0.536 | 0.557 | 0.673 | 0.652 |
|  | Asymp. Sig. (2-tailed) | 0.534 | 0.884 | 0.969 | 0.937 | 0.916 | 0.756 | 0.789 |
| 54mg | N | 10 | 10 | 10 | 10 | 10 | 10 | 10 |
|  | Kolmogorov-Smirnov Z | 0.880 | 0.443 | 0.449 | 0.624 | 0.685 | 0.776 | 0.507 |
|  | Asymp. Sig. (2-tailed) | 0.421 | 0.990 | 0.988 | 0.831 | 0.736 | 0.583 | 0.960 |

**Table S30**

Primary Pharmacokinetic Parameters for Different Dosing Groups of Ampiroxicam Following Single Oral Administration Homogeneity of Variances Test

|  | Levene Statistic | df_1_ | df_2_ | P value |
| --- | --- | --- | --- | --- |
| T_1/2_ | 2.011 | 2 | 27 | 0.153 |
| CL | 3.774 | 2 | 27 | 0.036 |
| V | 2.176 | 2 | 27 | 0.133 |
| C_max_ | 4.792 | 2 | 27 | 0.017 |
| AUC _0-216_ | 9.522 | 2 | 27 | 0.001 |
| AUC_0-∞_ | 9.402 | 2 | 27 | 0.001 |
| MRT | 0.447 | 2 | 27 | 0.644 |

**Table S31**

Nonparametric Test Results (Friedman Test) for Tmax Following Single Oral Administration of Ampiroxicam at Different Doses

| N | Chi-Square | df | Asymp. Sig |
| --- | --- | --- | --- |
| 10 | 0.560 | 2 | 0.756 |

**Table S32**

Data from 15 male subjects across three dosage groups in a single-dose administration study of Ampiroxicam

| NO. | Gender | Age | Height | Weight | Temperature | Respiratory  Rate | Heart  Rate | Systolic  Pressure | Diastolic  Pressure | RBC | Hb | WBC | PLT | ALT | AST | ALP | Cr | BUN | Urinalysis | Electrocardiogram |
| --- | --- | --- | --- | --- | --- | --- | --- | --- | --- | --- | --- | --- | --- | --- | --- | --- | --- | --- | --- | --- |
|  | Male/Female | year | cm | kg | ℃ | time/min | time/  min | mmHg | mmHg | 10^12^/L | g/L | 10^9^/L | 10^9^/L | U/L | U/L | U/L | μmol/L | mmol/L |  |  |
| 1 | Male | 20 | 165 | 52 | 36.4 | 18 | 64 | 120 | 80 | 5.45 | 152 | 7.7 | 240 | 14 | 22 | 76 | 78 | 4.97 | Normal | Normal |
| 2 | Male | 19 | 168 | 54 | 36.7 | 18 | 64 | 115 | 75 | 4.84 | 152 | 6.4 | 209 | 12 | 19 | 112 | 66 | 4.19 | Normal | Normal |
| 3 | Male | 19 | 170 | 56 | 36.0 | 18 | 60 | 115 | 60 | 5.09 | 150 | 9.1 | 219 | 17 | 18 | 81 | 72 | 4.05 | Normal | Normal |
| 4 | Male | 25 | 165 | 57 | 36.9 | 20 | 62 | 120 | 70 | 5.17 | 161 | 8.2 | 236 | 15 | 17 | 78 | 76 | 4.39 | Normal | Normal |
| 5 | Male | 21 | 166 | 59 | 36.1 | 20 | 64 | 120 | 70 | 5.02 | 146 | 5.8 | 295 | 18 | 23 | 54 | 68 | 4.89 | Normal | Normal |
| 6 | Male | 24 | 162 | 60 | 36.5 | 18 | 80 | 110 | 80 | 5.39 | 153 | 8.6 | 255 | 38 | 19 | 106 | 73 | 4.35 | Normal | Normal |
| 7 | Male | 26 | 170 | 60 | 36.5 | 18 | 60 | 110 | 60 | 4.28 | 136 | 6.0 | 214 | 13 | 15 | 84 | 60 | 4.15 | Normal | Normal |
| 8 | Male | 24 | 172 | 62 | 36.3 | 20 | 64 | 110 | 70 | 4.34 | 131 | 4.1 | 272 | 7 | 11 | 67 | 61 | 3.49 | Normal | Normal |
| 9 | Male | 24 | 171 | 64 | 36.2 | 18 | 74 | 120 | 70 | 5.07 | 156 | 7.5 | 208 | 21 | 16 | 81 | 71 | 4.7 | Normal | Normal |
| 10 | Male | 22 | 180 | 65 | 36.2 | 18 | 64 | 120 | 80 | 5.02 | 144 | 4.1 | 169 | 11 | 10 | 53 | 76 | 5.96 | Normal | Normal |
| 11 | Male | 20 | 175 | 67 | 36.1 | 18 | 72 | 110 | 80 | 4.69 | 135 | 7.6 | 198 | 20 | 14 | 80 | 64 | 3.51 | Normal | Normal |
| 12 | Male | 24 | 172 | 68 | 36.2 | 20 | 72 | 120 | 80 | 5.24 | 155 | 6.6 | 237 | 31 | 25 | 75 | 87 | 4.66 | Normal | Normal |
| 13 | Male | 22 | 180 | 74 | 36.3 | 18 | 72 | 110 | 70 | 4.82 | 152 | 4.6 | 150 | 15 | 12 | 60 | 62 | 6.44 | Normal | Normal |
| 14 | Male | 21 | 177 | 75 | 36.0 | 20 | 72 | 120 | 70 | 5.02 | 145 | 6.0 | 187 | 26 | 24 | 106 | 77 | 3.66 | Normal | Normal |
| 15 | Male | 20 | 180 | 75 | 36.0 | 20 | 60 | 115 | 65 | 4.88 | 151 | 7.5 | 235 | 21 | 15 | 68 | 67 | 5.35 | Normal | Normal |
| SD |  | 2.28 | 5.87 | 7.45 | 0.27 | 1.01 | 6.18 | 4.58 | 7.02 | 0.33 | 8.45 | 1.57 | 37.85 | 8.09 | 4.70 | 17.99 | 7.53 | 0.85 |  |  |
| Mean |  | 22.07 | 171.53 | 63.20 | 36.29 | 18.80 | 66.93 | 115.67 | 72.00 | 4.95 | 147.93 | 6.65 | 221.60 | 18.60 | 17.33 | 78.73 | 70.53 | 4.58 |  |  |
| Normal Values |  |  |  |  |  |  |  |  |  | 4.09-  5.74 | 131-172 | 3.97-  9.15 | 85-  303 | 5-40 | 8-40 | 53-  128 | 44-80 | 2.9-8.2 |  |  |

**Table S33**

Data from 15 female subjects across three dosage groups in a single-dose administration study of Ampiroxicam

| NO. | Gender | Age | Height | Weight | Temperature | Respiratory  Rate | Heart  Rate | Systolic  Pressure | Diastolic  Pressure | RBC | Hb | WBC | PLT | ALT | AST | ALP | Cr | BUN | Urinalysis | Electrocardiogram |
| --- | --- | --- | --- | --- | --- | --- | --- | --- | --- | --- | --- | --- | --- | --- | --- | --- | --- | --- | --- | --- |
|  | Male/Female | year | cm | kg | ℃ | time/min | time/  min | mmHg | mmHg | 10^12^/L | g/L | 10^9^/L | 10^9^/L | U/L | U/L | U/L | μmol/L | mmol/L |  |  |
| 16 | Female | 24 | 150 | 50 | 36.0 | 20 | 60 | 110 | 65 | 4.4 | 128 | 7.6 | 230 | 11 | 17 | 55 | 45 | 3.23 | Normal | Normal |
| 17 | Female | 19 | 152 | 50 | 36.8 | 20 | 72 | 115 | 60 | 4.4 | 131 | 5.5 | 203 | 16 | 20 | 80 | 55 | 6.54 | Normal | Normal |
| 18 | Female | 21 | 152 | 50 | 36.0 | 16 | 68 | 90 | 60 | 4.53 | 130 | 7.4 | 179 | 13 | 18 | 56 | 62 | 5.13 | Normal | Normal |
| 19 | Female | 23 | 155 | 50 | 36.0 | 20 | 60 | 120 | 70 | 4.63 | 129 | 5.0 | 243 | 24 | 20 | 53 | 48 | 4.06 | Normal | Normal |
| 20 | Female | 21 | 158 | 50 | 36.2 | 18 | 60 | 105 | 65 | 4.23 | 116 | 9.0 | 296 | 32 | 27 | 59 | 55 | 4.11 | Normal | Normal |
| 21 | Female | 21 | 160 | 50 | 36.0 | 18 | 64 | 110 | 60 | 4.31 | 121 | 6.3 | 222 | 14 | 22 | 56 | 58 | 5.73 | Normal | Normal |
| 22 | Female | 23 | 160 | 50 | 36.3 | 16 | 72 | 105 | 70 | 4.01 | 115 | 4.4 | 175 | 14 | 17 | 58 | 49 | 3.72 | Normal | Normal |
| 23 | Female | 23 | 160 | 51 | 36.9 | 18 | 60 | 115 | 75 | 4.62 | 132 | 4.8 | 179 | 8 | 19 | 54 | 56 | 3.41 | Normal | Normal |
| 24 | Female | 19 | 161 | 51 | 36.4 | 18 | 60 | 100 | 80 | 4.71 | 138 | 7.0 | 253 | 10 | 22 | 62 | 59 | 4.31 | Normal | Normal |
| 25 | Female | 19 | 163 | 52 | 36.4 | 18 | 72 | 105 | 65 | 4.25 | 128 | 8.6 | 219 | 11 | 16 | 76 | 46 | 3.69 | Normal | Normal |
| 26 | Female | 22 | 163 | 53 | 36.6 | 20 | 72 | 130 | 80 | 4.00 | 122 | 5.4 | 183 | 11 | 18 | 58 | 49 | 3.12 | Normal | Normal |
| 27 | Female | 18 | 168 | 54 | 36.0 | 20 | 72 | 120 | 70 | 4.68 | 129 | 5.5 | 251 | 12 | 26 | 79 | 58 | 4.22 | Normal | Normal |
| 28 | Female | 20 | 168 | 55 | 36.1 | 20 | 60 | 110 | 70 | 4.44 | 125 | 4.4 | 244 | 8 | 17 | 54 | 59 | 3.26 | Normal | Normal |
| 29 | Female | 22 | 166 | 56 | 36.0 | 18 | 68 | 110 | 70 | 3.82 | 114 | 4.4 | 208 | 12 | 22 | 55 | 52 | 3.42 | Normal | Normal |
| 30 | Female | 20 | 162 | 57 | 36.6 | 20 | 76 | 90 | 60 | 4.14 | 119 | 5.9 | 188 | 12 | 10 | 54 | 52 | 3.38 | Normal | Normal |
| SD |  | 1.81 | 5.63 | 2.46 | 0.32 | 1.45 | 6.01 | 10.72 | 6.76 | 0.27 | 7.03 | 1.52 | 35.05 | 6.30 | 4.17 | 9.50 | 5.26 | 1.00 |  |  |
| Mean |  | 21.00 | 159.87 | 51.93 | 36.29 | 18.67 | 66.40 | 109.00 | 68.00 | 4.34 | 125.13 | 6.08 | 218.20 | 13.87 | 19.40 | 60.60 | 53.53 | 4.09 |  |  |
| Normal Values |  |  |  |  |  |  |  |  |  | 3.68-5.13 | 113-  151 | 3.69-  9.16 | 101-  320 | 5-40 | 8-40 | 53-  128 | 44-80 | 2.9-8.2 |  |  |

**Table S34**

Data from 10 subjects in a multiple-dose administration study of Ampiroxicam across various dosage groups

| NO. | Gender | Age | Height | Weight | Body  Temperature | Respiratory  Rate | Heart  Rate | Systolic  Pressure | Diastolic  Pressure | RBC | Hb | WBC | PLT | ALT | AST | ALP | Cr | BUN | Urinalysis | Electrocardiogram |
| --- | --- | --- | --- | --- | --- | --- | --- | --- | --- | --- | --- | --- | --- | --- | --- | --- | --- | --- | --- | --- |
|  | Male/Female | year | cm | kg | ℃ | time/min | time/  min | mmHg | mmHg | 10^12^/L | g/L | 10^9^/L | 10^9^/L | U/L | U/L | U/L | μmol/L | mmol/L |  |  |
| 31 | Male | 20 | 175 | 65 | 36.2 | 18 | 72 | 105 | 70 | 4.38 | 135 | 8.6 | 260 | 19 | 18 | 60 | 71 | 3.75 | Normal | Normal |
| 32 | Male | 20 | 184 | 74 | 36.2 | 18 | 68 | 125 | 80 | 4.85 | 138 | 4.0 | 203 | 16 | 21 | 110 | 67 | 5.06 | Normal | Normal |
| 33 | Male | 21 | 165 | 65 | 36.3 | 20 | 70 | 105 | 70 | 4.59 | 132 | 6.7 | 219 | 12 | 18 | 58 | 86 | 4.52 | Normal | Normal |
| 34 | Male | 21 | 180 | 65 | 36.4 | 18 | 68 | 120 | 80 | 5.12 | 151 | 4.6 | 269 | 19 | 20 | 64 | 63 | 3.22 | Normal | Normal |
| 35 | Male | 22 | 185 | 70 | 36.0 | 20 | 72 | 100 | 20 | 4.7 | 144 | 7.5 | 286 | 11 | 13 | 66 | 91 | 4.77 | Normal | Normal |
| 36 | Female | 22 | 170 | 68 | 36.2 | 20 | 72 | 130 | 80 | 4.42 | 124 | 6.3 | 257 | 11 | 11 | 73 | 74 | 4.5 | Normal | Normal |
| 37 | Female | 22 | 165 | 55 | 36.4 | 20 | 66 | 90 | 60 | 3.83 | 122 | 8.2 | 252 | 28 | 26 | 77 | 68 | 6.05 | Normal | Normal |
| 38 | Female | 23 | 164 | 56 | 36.3 | 20 | 66 | 90 | 60 | 4.18 | 121 | 4.2 | 250 | 35 | 40 | 67 | 61 | 3.81 | Normal | Normal |
| 39 | Female | 22 | 163 | 63 | 36.4 | 18 | 72 | 100 | 60 | 4.58 | 129 | 5.8 | 306 | 23 | 16 | 64 | 52 | 3.34 | Normal | Normal |
| 40 | Female | 20 | 163 | 52 | 36.4 | 20 | 60 | 100 | 60 | 3.82 | 113 | 8.7 | 315 | 9 | 17 | 76 | 79 | 6.74 | Normal | Normal |
| SD | Male | 0.84 | 8.17 | 4.09 | 0.15 | 1.10 | 2.00 | 10.84 | 25.10 | 0.28 | 7.58 | 1.94 | 34.98 | 3.78 | 3.08 | 21.70 | 12.24 | 0.76 |  |  |
| Mean | Male | 20.80 | 177.8 | 67.80 | 36.22 | 18.80 | 70.0 | 111.00 | 64.00 | 4.73 | 140.0 | 6.28 | 247.4 | 15.40 | 18.00 | 71.60 | 75.60 | 4.26 |  |  |
| SD | Female | 1.10 | 2.92 | 6.53 | 0.09 | 0.89 | 5.02 | 16.43 | 8.94 | 0.34 | 5.81 | 1.83 | 31.76 | 11.10 | 11.42 | 5.68 | 10.66 | 1.46 |  |  |
| Mean | Female | 21.80 | 165.0 | 58.80 | 36.34 | 19.60 | 67.2 | 102.0 | 64.00 | 4.17 | 121.8 | 6.64 | 276.0 | 21.20 | 22.00 | 71.40 | 66.80 | 4.89 |  |  |
