# Supplementary figures and images for: Exploring the pharmacokinetics and tolerability of cyclooxygenase inhibitor ampiroxicam: a phase I study on single and multiple oral doses
Source: Front Pharmacol. 2024 Jun 21;15:1429971. doi: 10.3389/fphar.2024.1429971 (PMC11224448; doi:10.3389/fphar.2024.1429971)

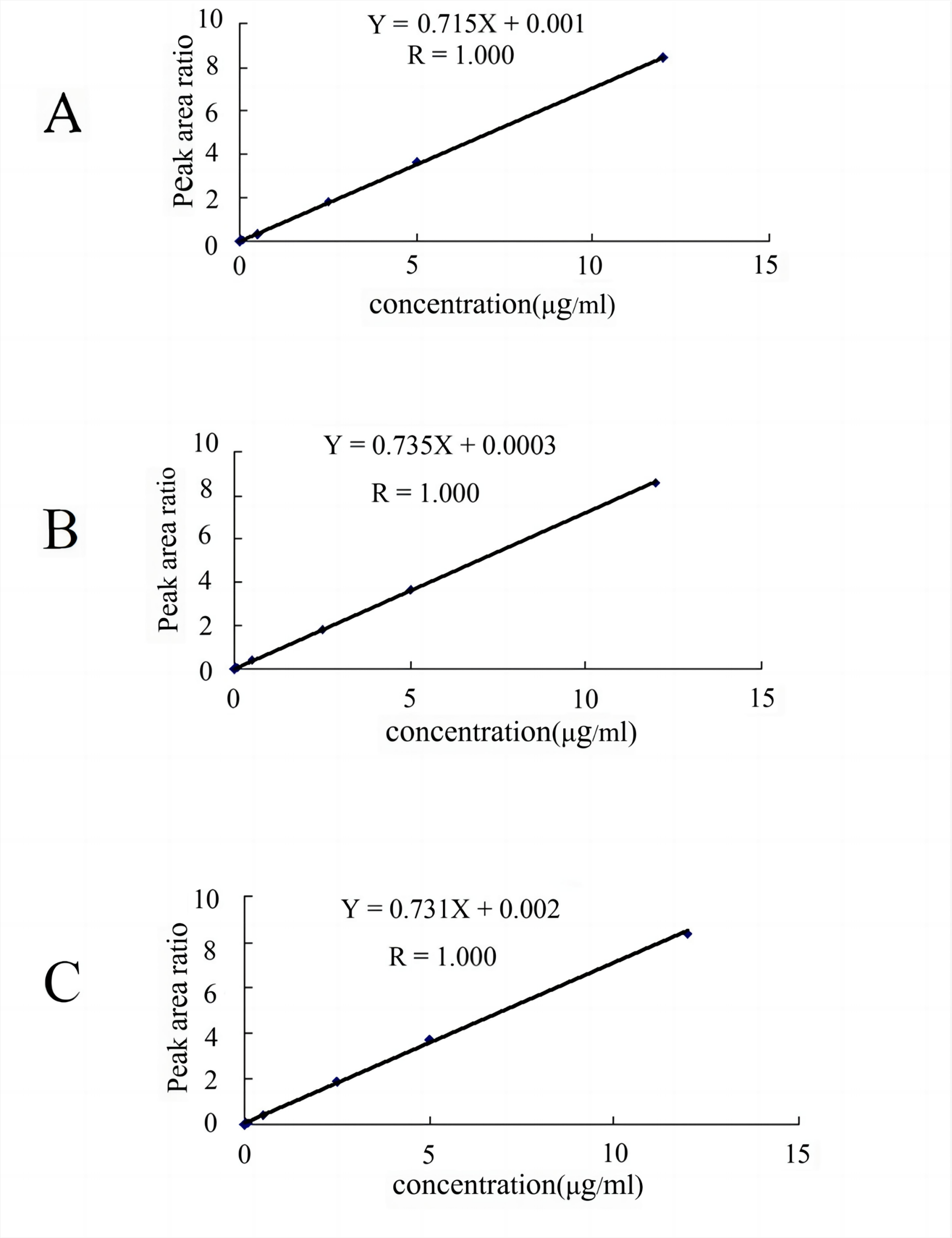

Supplement: Supplementary file 2 [file Image3.TIF]

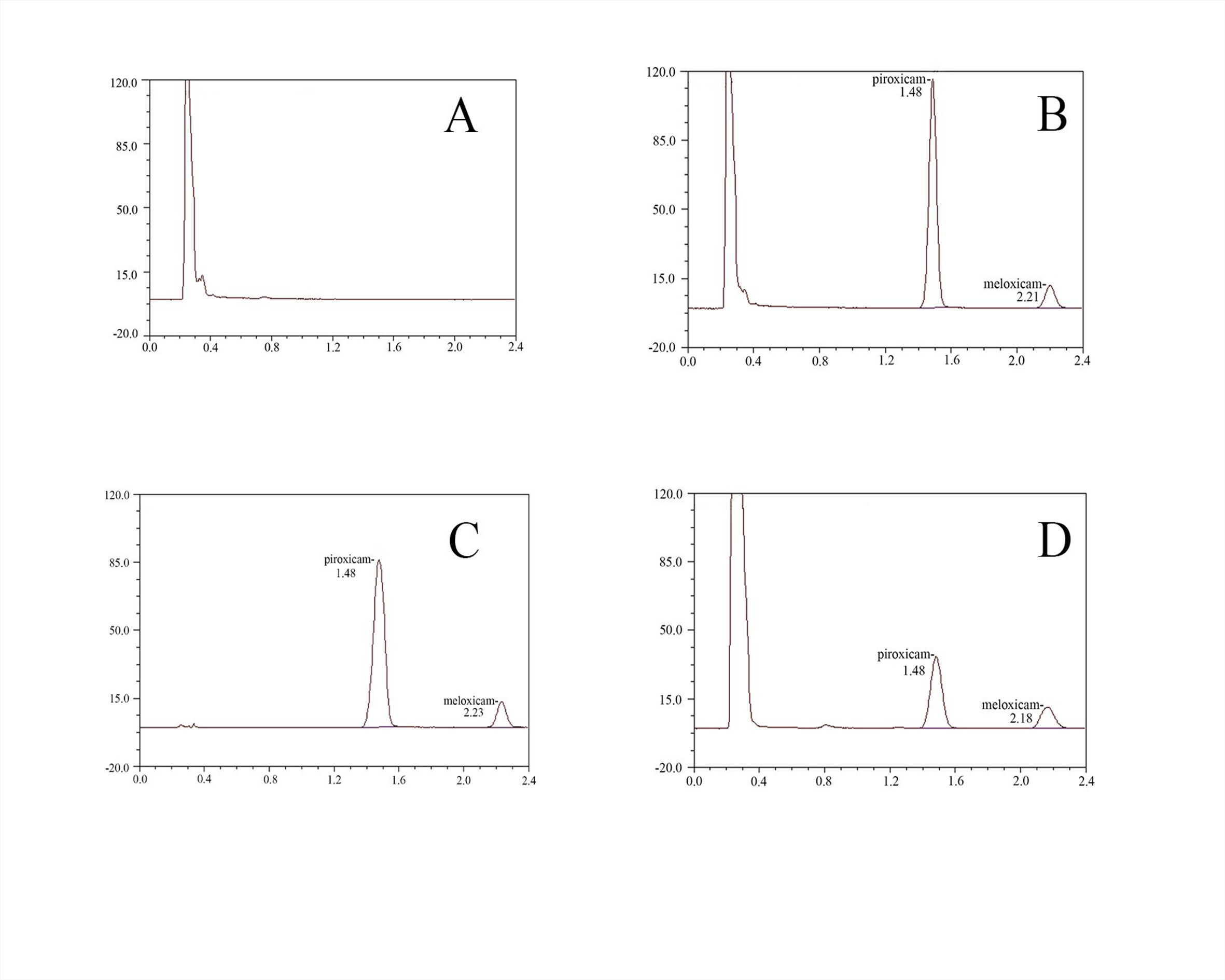

Supplement: Supplementary file 3 [file Image2.TIF]

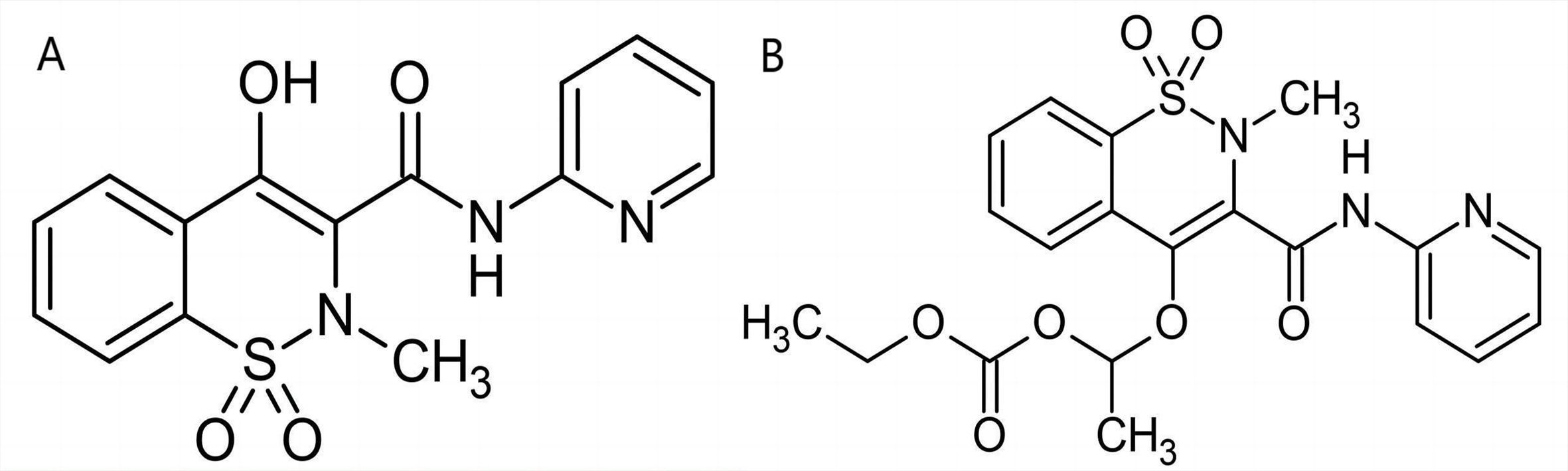

Supplement: Supplementary file 4 [file Image1.TIF]
